# Stable Air Plastron Prolongs Biofluid Repellency of Submerged Superhydrophobic Surfaces

**DOI:** 10.1021/acs.langmuir.4c04259

**Published:** 2025-01-15

**Authors:** Mohammad Awashra, Seyed Mehran Mirmohammadi, Lingju Meng, Sami Franssila, Ville Jokinen

**Affiliations:** School of Chemical Engineering, Department of Chemistry and Materials Science, Aalto University, Tietotie 3 Espoo 02150, Finland

## Abstract

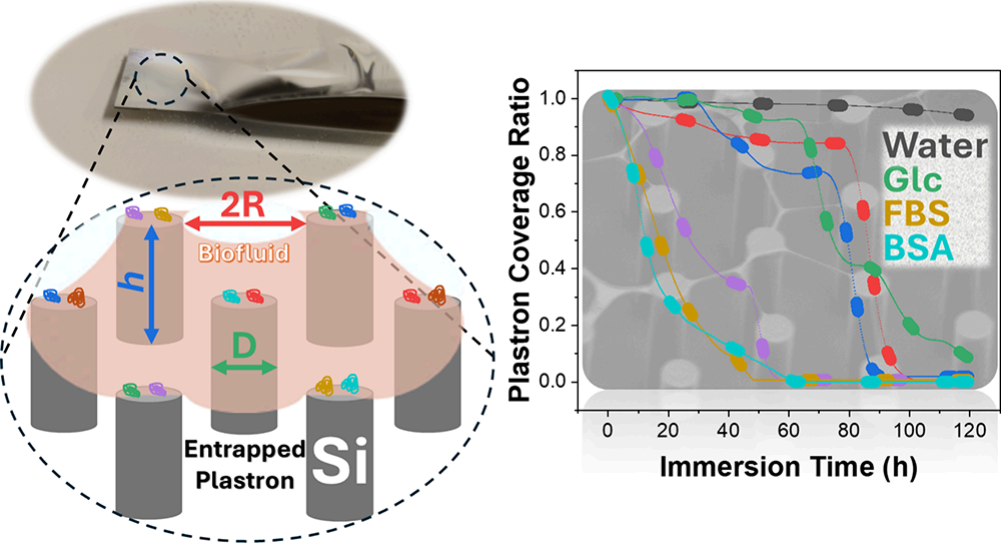

Superhydrophobic surfaces find applications in numerous biomedical scenarios, requiring the repellence of biofluids and biomolecules. Plastron, the trapped air between a superhydrophobic surface and a wetting liquid, plays a pivotal role in biofluid repellency. A key challenge, however, is the often short-lived plastron stability in biofluids and the lack of knowledge surrounding it. Plastron stability refers to the duration for which a surface remains in the Cassie state before transitioning to the fully wetting Wenzel state. Here, a submersion test with real-time optical monitoring is used to determine the plastron lifetime of different superhydrophobic surfaces upon immersion in various biofluids. We find that biofluids of all types exhibit shorter plastron lifetimes compared to pure water, which is attributed to their lower surface tension and biomolecular adsorption through hydrophobic–hydrophobic interactions. Proteins and glucose are identified as the major contributors to plastron dissipation in fetal bovine serum-based biofluids. Plastron minimizes the solid–liquid interface, reducing biomolecular adsorption, making its stability crucial for biofluid repellence. Thus, the effects of surface texture, feature size, Cassie solid fraction, Wenzel dimensionless roughness, and surface chemistry on plastron stability are investigated. Our key findings indicate that prolonged plastron stability and thus enhanced biofluid repellency are achieved through a combination of larger plastron volumes, increased Wenzel roughness degrees, greater Cassie solid fractions, and smaller feature sizes. We demonstrate that with optimized parameters, our surface design can maintain plastron stability and sustain a consistent solid–liquid area fraction for over 120 h in complex biofluids containing high levels of protein and glucose, underscoring a robust design for long-term use in biomedical and antifouling applications. This research is essential for advancing the design of superhydrophobic surfaces that effectively resist biofouling in diverse medical and engineering settings.

## Introduction

Superhydrophobic (SHB) surfaces repel water and are often defined as having contact angles higher than 150° and sliding angles lower than 10°.^1−4^ Superhydrophobicity is achieved when a low surface energy is combined with a micro/nanoscale roughness.^5^ There are some scenarios where SHB surfaces are completely submerged in liquids, such as in drag-reducing coatings on the bottom of a ship or an underwater sensor.^6,7^ In other scenarios, surfaces may be entirely surrounded by liquid, as in the case of water or blood flowing through a tube or a capillary. In such cases, the contact angle- and sliding angle-based definition of superhydrophobicity is not applicable since there is no discrete droplet.^8−11^ Cassie and Baxter suggested that the liquid comes into contact with only a fraction of the solid surface (*i.e*., roughness summits or initial solid fraction *f*_*o*_) and the rest of the liquid is positioned on entrapped air.^12^ The trapped layer of air is called plastron (sometimes also referred to as entrapped air layer, air pockets, air bubbles, or air cushion),^13^ and it is crucial for superhydrophobicity. Losing the plastron means an irreversible transition from the Cassie to Wenzel wetting state, where the liquid will come into full contact with the solid surface (*f* = 1).^14^ To make a surface as superhydrophobic as possible, the solid fraction at the Cassie state (*f*_c_) should be kept as small as possible.^8^ Hence, in many applications, it is crucial for SHB surfaces to possess the capability of not only trapping air (Cassie state) but also maintaining its presence over an extended duration (long plastron lifetime).^15^ The energy barrier of Cassie to Wenzel transition, and thus the stability of the plastron, depends on the type of SHB surface (surface chemistry and morphology), and the wetting liquid surface tension and its possible adsorptive or reactive components, as well as the surrounding environmental parameters (*i.e*., pressure, temperature, flow rate, etc.).^11,16^ For each of these parameters, there is a threshold at which the plastron is lost.

In some biomedical technologies, such as antibiofouling, biosensors, medical and blood contacting devices, cell-trapping, and biological assays, surfaces have to be fully immersed in biofluids that are more complex than water (e.g., cell media, blood, and urine).^17−20^ Biofluids can contain cells, proteins, and other biomolecules that affect their properties.^21^ Due to their plastron, SHB surfaces are gaining escalating interest as a promising strategy for long-term antibiofouling and they have been used in many biomedical applications.^17,22−25^ While most SHB surfaces are initially biofluid-repellent, biomolecular adsorption and biofluids’ lower surface tension compared to water are two factors that can significantly affect their air plastron durability and, subsequently, their performance as biofluid-repellent surfaces.^26−29^ Toes et al.^30^ highlighted that, *in vivo*, a superhydrophobic modified polytetrafluoroethylene artificial blood vessel did not enhance anticoagulant performance. Conversely, this led to increased platelet deposition in an extracorporeal circulation test. Li et al.^31^ used a carbon nanofiber superhydrophobic surface as a fast clotting hemostatic agent. When rolling a blood droplet over the surface, the authors observed deposited residual fibrin fibers that remained on the surface. Our previous work showed that the plastron of a black Si (nanograss structure with 5–10 nm spike size and 1.4 μm height) surface is immediately lost when exposed to nucleic acid detection solution and it was concluded that this is a result of the extremely low surface tension and high biomolecular adsorption affinity onto the hydrophobic coating.^32^ There have been several other techniques, such as hydrogel coating and slippery liquid infused porous surfaces, that have been applied as biofluid-repellent materials. However, such surface modification cannot certainly avoid biological contamination in long-term applications.^17,22^

Previous studies have explored the design of superhydrophobic surfaces for maintaining air plastrons in water or simple aqueous solutions.^11,33^ However, these approaches typically fail in complex biofluids due to rapid biomolecular adsorption, which compromises plastron stability within minutes to hours.^34,35^ Proteins generally exhibit nonspecific adsorption onto hydrophobic surfaces through hydrophobic–hydrophobic interactions, leading to destabilization of the plastron and significantly limiting the long-term biofluid repellency of superhydrophobic surfaces.^36,37^ Despite growing interest in the biomedical applications of superhydrophobic surfaces, research examining their performance under full immersion in biofluids remains scarce. Existing studies predominantly focus on protein or cell adsorption, blood coagulation, or general antifouling properties of the SHB surface compared to flat surfaces, rather than exploring the durability of the air plastron or providing actionable design criteria to address plastron dissipation. For instance, Zhang et al.^34^ reported that an SHB Ti surface has minimum protein and blood cell adsorption compared to flat Ti surfaces, where the SHB surface and protein show a repulsive interaction as the distance between them is getting smaller. However, their study did not evaluate plastron durability under immersion.^19^ On the other hand, studies that do investigate plastron lifetime in biofluids often report very short lifetimes and are limited in scope, and this type of study is even rarer. Tesler et al.^19^ reported a plastron lifetime exceeding 208 days in water and demonstrated short-term blood repellency upon repeated rapid 1 s immersions in blood. Wang et al.^35^ studied the impact of bovine serum albumin (BSA) protein on the longevity of the air plastron of a certain SHB surface and found that the plastron lifetime was shorter for higher BSA concentrations due to higher protein adsorption and lower surface tension. However, their findings were limited to plastron lifetimes of 5–10 min, and they did not explore the effects of other biomolecules or propose strategies to enhance plastron stability. In contrast, our study achieves plastron stability lasting over 120 h, even in biofluids with high protein and glucose concentrations. This is accomplished through a unique combination of surface design parameters including optimized feature size, solid fraction, and surface chemistry. These findings represent a significant advancement in the development of biofluid-repellent surfaces for long-term biomedical applications.

To design a biofluid-repellent SHB surface for biomedical applications, Cassie solid fraction *f*_c_ should be minimized, and the plastron lifetime should be maximized when immersed in a biofluid. These primarily depend on the shape of the liquid–air interface, which is directly affected by the SHB surface roughness and surface chemistry, as well as the biofluid compositions and their concentration.^5^ Kanungo et al.^38^ showed that superhydrophobicity increases as roughness increases. However, do the biofluid-repellent properties always increase as the superhydrophobicity increases? Luo et al.^22^ note that biofluid-repellent properties of SHB surfaces have contradicting results, and so, there is a need for a systematic study that gives a unified understanding of SHB surface interaction with biofluids. Studying the plastron lifetime of fully submerged SHB surfaces in various biofluids is essential to address the extent and durability of SHB surface biofluid repellency. Most existing research primarily focuses on studying the wettability of biofluid droplets on SHB surfaces instead of their full immersion. Here, we aim to systematically examine the plastron lifetime of SHB surfaces when fully submerged in biofluids. Furthermore, we explore the impact of SHB surface morphology and chemistry on the plastron lifetime to contribute to the design of effective and durable biofluid-repellent SHB surfaces. Our research helps in enhancing the long-term stability of SHB surfaces applied in the biomedical field.

## Experimental Methods

### Surface Fabrication

Five different types of surfaces were fabricated in this study: Si micropillars with varying pillar sizes, heights, and solid fractions, black Si, Si nanopillars, copper-polydimethylsiloxane (Cu-PDMS) composite surface,^39^ and PDMS micropillars. See Supporting Information Section 1.1. The geometry parameters of the different Si micropillared surfaces fabricated are shown in Table S1.

### Biofluid Preparation

The used biofluids contained different concentrations of bovine serum albumin (BSA), fetal bovine serum (FBS), and glucose, and the solvents were either deionized water or RPMI 1640 cell medium (Gibco). Table S2 shows all of the prepared biofluids. BSA powder (Fisher BioReagents) was dissolved in the solvent by stirring for 10 min at room temperature. FBS (Gibco) and glucose (200 mg mL^–1^, Gibco) solutions were pipetted into RPMI 1640 media (Gibco) in a biosafety laminar hood. All biofluids are supplemented with 1% penicillin-streptomycin (10,000 U/mL, Gibco). A volume of 70 mL of each prepared biofluid, corresponding to a liquid height of 4.0 cm (significantly exceeding the typical biofluid height required in most biomedical applications) was dispensed into a sterilized 100 mL glass beaker. The beaker was sealed to inhibit evaporation, ensuring a constant hydrostatic pressure of approximately 400 Pa throughout the experiments.

### Surface Coatings

Three surface chemistries were prepared to study the effect of surface coating on plastron lifetime: plasma-deposited fluoropolymer film coating, 1H,1H,2H,2H-perfluorododecyltrichloro silane (PDTS) self-assembled monolayer, and PDMS polymer. The fluoropolymer coating on black Si was prepared as demonstrated earlier in the Surface Fabrication Section. The PDTS coating was applied on a black Si surface as the following: A 4 in. black Si wafer was treated with oxygen plasma and placed in a glass Petri dish, and then, a few milligrams of PDTS silane (Glpbio) were introduced into a small holder within the Petri dish. The Petri dish was then capped and placed on a hot plate at 100 °C for 2 h. The silane-coated black Si was then ready for use. The PDMS 10 μm pillars were prepared as described earlier in the Surface Fabrication Section. All three surfaces were immersed in 100 mg mL^–1^ BSA and 2 mg mL^–1^ glucose dissolved in FBS, and the plastron measurements were then performed.

### Contact Angle and Surface Tension Measurements

The dynamic advancing and receding contact angles of water and the biofluids were measured by using the needle-in sessile drop technique (THETA, Biolin Scientific). The advancing contact angle was measured from a 2 to 5 μL droplet size, and the receding contact angle was measured by the reducing droplet size from 5 to 0 μL with a droplet rate of 0.1 μL s^–1^. The surface tension measurements of water and all biofluids were performed optically by using the pendant drop method with a droplet size of 5 μL (THETA, Biolin Scientific). All experiments were performed in triplicate, and the reported value is in the form mean ± standard deviation.

### Optical Monitoring Setup

**Figure 1:**
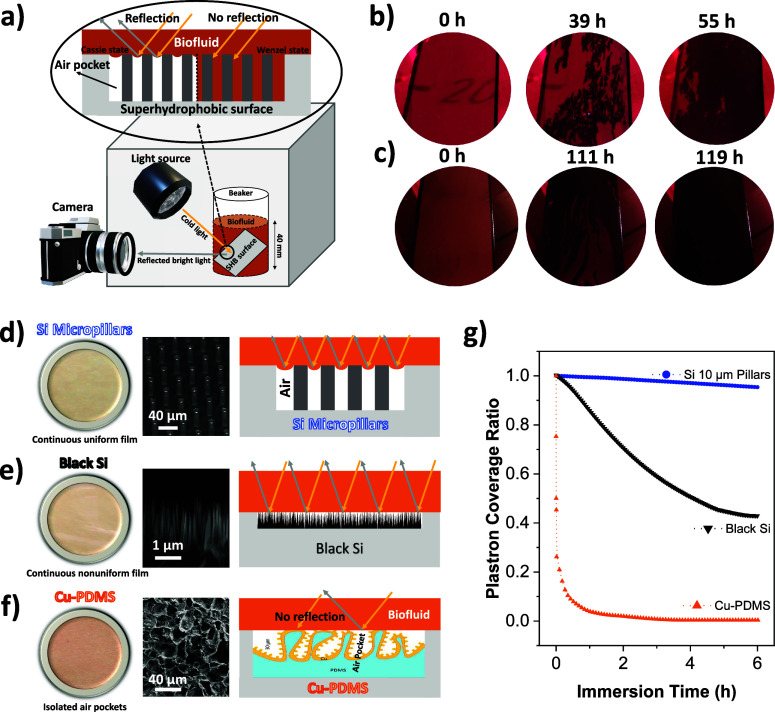
Optical monitoring setup and the plastron shape observation. (a) Schematic of the optical setup used for plastron monitoring in this study. Time-lapse images of an SHB surface with 10 μm Si micropillars submerged in (b) 10% FBS supplemented with 50 mg mL^–1^ BSA and (c) 1.25 mg mL^–1^ glucose, both dissolved in RPMI 1640 medium. The plastron lifetime and shape for three distinct superhydrophobic textures submerged in protein solutions are shown: optical images of the plastron (left), an SEM image of the surface (middle), and corresponding schematic representations (right) for (d) Si micropillars, (e) black Si, and (f) Cu-PDMS.^40^ (g) Plastron stability over time for the three surfaces immersed in a solution containing 20 mg mL^–1^ BSA.

Plastron coverage was measured using an optical monitoring setup utilizing a light source and a camera. Similar setups were used by Bobji et al.,^15^ Poetes et al.,^16^ Wu et al.,^18^ and Wang et al.^35^ A schematic illustration of the used experimental setup is shown in Figure 1a.

A 3 cm × 3 cm chip of each prepared SHB surface was fully submerged in the studied biofluid. The plastron dissipation was observed using a video camera (Canon 7D + Canon Macro lens EF-S 60 mm, 5184 × 3456 pixels) and a cold light illumination (Godox LED126) that is directed toward the SHB surface at a fixed position and angle.^41^ Good care was taken to ensure that these parameters remained unchanged over time. The experimental setup is contained in a black box to minimize light scattering and interferences from other light sources. The temperature of the system was fixed at 21 ± 1 °C. Liquid evaporation was minimized by sealing the liquid container.

Light that is reflected (total internal reflection) from the liquid–air interface of the plastron has a bright silver-like color, which was identified as the Cassie state. On the other hand, if the plastron is not present, light is not reflected from the solid–liquid interface, resulting in less reflected light and a darker appearance, which was identified as the Wenzel state. The plastron was first recorded continuously for the first 10 min, and then, one image was taken every 10 min for about 120 h or until the plastron completely dissipates. About 50 plastron measurement experiments were performed, and 50 000 data points were collected in this study. Figure 1b,c shows a sample images series of 10 μm Si pillars submerged in two different biofluids captured using the setup at varied immersion times.

### Image Analysis

All images taken for one experiment (700–1000 images) were analyzed using ImageJ software.^42^ The plastron coverage of the 9 cm^2^ area of each surface was measured for all the images. The background noise was eliminated in each image by subtracting the measurement of the surface in the Wenzel state. The plastron coverage ratio was calculated for each data point by using eq 1. A moving average (*n* = 5) was applied to the data to smooth the plastron coverage curves. The solid–liquid area fraction is calculated from the plastron coverage ratio and Cassie solid fraction (*f*_0_) by using eq 2
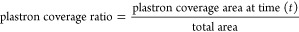
1
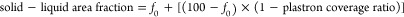
2

## Results and Discussion

### Surface Topography and Wettability

Figure S1 shows the SEM images of the SHB surfaces studied. The Si nanopillars (Figure S1a) were fabricated by using electron beam lithography. The black Si surface (Figure S1b) utilized a maskless plasma etching method. The Cu-PDMS composite surface (Figure S1c,d) was fabricated by a facile and low-cost method based on polymer replication,^39^ while Si micropillars (Figure S1e,f) were achieved using photolithography followed by a deep plasma etching technique. PDMS pillars were also fabricated. The fluoropolymer-coated Si micropillared surfaces were fabricated with different pillar sizes (2–50 μm), solid fractions (2.5–22.7%), and pillar heights (3–40 μm). The superhydrophobicity of all surfaces was characterized by using goniometry (Figure S2). Two control surfaces were used: a hydrophobic fluoropolymer-coated smooth Si surface (HB ref) that has a water advancing contact angle of 110° and a receding contact angle of 80° and a hydrophilic noncoated smooth Si surface (HL ref) with a water advancing contact angle of 33° and a receding contact angle of 16°. All SHB surfaces have water advancing and receding contact angles greater than 150° except for the PDMS pillar surface that has a receding contact angle of about 135°. Black Si has the highest advancing contact angle and the lowest contact angle hysteresis. The Si micropillared surface dynamic contact angles were 167° (Adv) and 159° (Rec).

### Plastron Dissipation Mechanisms

The plastron lifetime is defined here as the time needed for the air trapped on the SHB surface to fully dissipate and the solid–liquid area fraction becomes close to 1.0 (*i.e.*, the surface to become black or nonreflective). Before addressing the plastron lifetime of superhydrophobic surfaces in biofluids, we first examine the plastron shape on various surface textures and its dissipation mechanisms. Figure 1d–g shows the plastron shape and lifetime of three surface textures (Si micropillars, black Si nanograss, and micronano-hierarchical Cu-PDMS) immersed in 20 mg mL^–1^ BSA solution. The plastron on the Si micropillared surface maintained full plastron coverage for more than 6 h, and the plastron coverage on black Si was about 40% after 6 h, while the plastron of the Cu-PDMS surface fully dissipated in a matter of minutes. The shape and dissipation behavior of the trapped air strongly depended on the surface texture. This was observed in the data obtained from plastron lifetime experiments on the three surfaces. The plastron can be in the form of uniform or nonuniform continuous air film or in the form of isolated air pockets. The plastron of a Si micropillared surface is a single connected air film throughout the surface since the pillars are uniform and periodic with an open trench structure (Figure 1d). For the black Si, the plastron is in the form of nonuniform film because of the nanograss random structure (Figure 1e). If the air film on these surfaces is curved as one big spherical spot, then only one bright spot would be observed, in the optical images in Figure 1d,e, at the top of the entire surface as Bobji et al.^15^ suggested. However, there is a continuous bright film over the surface, which indicates that there are plenty of small bright spots throughout the liquid–air interface between the micropillars or nanograss. For the Cu-PDMS surface, separate bright spots were observed instead of a continuous bright film, which is due to the fact that this surface has random micro-roughness that forms isolated pockets covered with nanobumps, enabling the formation of small and separated air pockets, as shown in Figure 1f.

The plastron is dissipating mainly through two routes: First, by air diffusion into water. This route occurs on the three surfaces. Epstein and Plesset^43^ showed that a spherical bubble with a radius of 100 μm would require approximately 59 min to completely dissolve through diffusion in a saturated solution.^43^ The continuous film on our micropillared surfaces has a large air volume, measuring in the mm scale. Moreover, the stability of the liquid–air interface on this structure makes plastron take days to weeks to fully dissipate (Figure 1g, blue circles). The air film volume of nanograss black Si plastron is much lower than the micropillars, making its diffusion rate greater (Figure 1g, black triangle). The isolated air pockets on the Cu-PDMS surface, on the contrary, have very small volumes due to their disconnectedness, making its plastron lifetime less than 1 h (Figure 1g, orange pyramids). It is worth mentioning that the surface coating of the Cu-PDMS surface is not fluoropolymer, which can significantly affect its plastron lifetime.

The second plastron dissipation route, termed the air-pushing bubble mechanism, occurs when capillary forces and hydrostatic pressure (along with other factors such as protein adsorption) cause liquid infiltration into the microspaces, displacing air into larger bubbles that eventually detach due to buoyancy.^35,44^ In cases where a biofluid serves as the wetting liquid, the extent of the biomolecular adsorption on the solid surface and the fluid’s lower surface tension play crucial roles in determining the mechanism of plastron dissipation. The air-pushing bubble mechanism takes place on the pillared surface, where the formation of a big air bubble is observed. Sun et al.^45^ observed the same phenomenon, where they noticed a gradually growing air bubble on an underwater SHB surface. Similarly, an agglomeration of air as more pores transit into the Wenzel state is detected until the buoyancy of the air beats the air bubble adhesion forces to the solid surface and then detaches and floats in the bulk liquid (see Supporting Video 1). Moreover, Tuberquia et al.^46^ observed that as the Wenzel state becomes more dominant, the air is compacted into growing air pockets. In water, the stability of the liquid–air interface at the nanoscale roughness is significantly greater when compared to the microscale roughness (see eq 3 in the coming sections).^47^ However, under a biofluid, biomolecular adsorption on nanosurfaces can change this trend. More on this can be found in the Supporting Information (Section S2.1).

### Effect of Biofluid Composition on Plastron Lifetime

**Figure 2:**
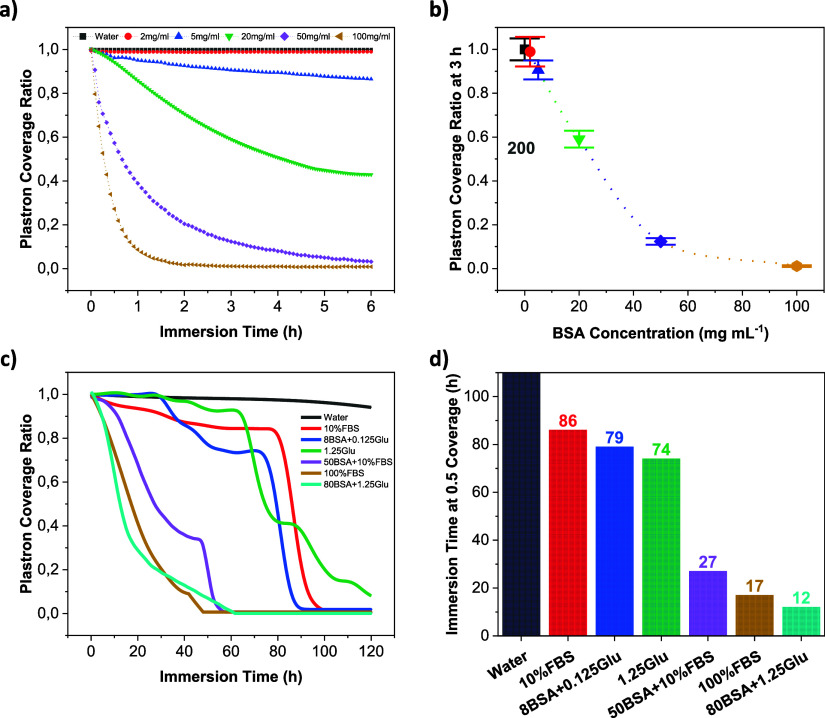
Effect of biofluid composition on plastron lifetime of superhydrophobic surfaces. (a) Plastron coverage ratio of a 9 cm^2^ black Si SHB surface vs its immersion time in solutions with different concentrations of BSA protein. (b) Effect of BSA concentration on the plastron dissipation of the black Si SHB surface after 3 h of complete immersion. (c) Plastron coverage ratio of a 9 cm^2^ 10 μm Si pillar SHB surface (solid fraction: 14.5%, pillar height: 15 μm) vs its immersion time in different biofluids. (d) Time needed for the 10 μm pillared SHB surface plastron to reach 50% of surface coverage when immersed in these biofluids.

Serum albumin is the most abundant protein in the blood plasma of all vertebrates and its concentration in human serum is ranging from 35 to 50 mg mL^–1^.^48^ It was decided to use bovine serum albumin (BSA) as a model protein in this study to investigate the effect of protein concentration on plastron lifetime. The BSA concentrations in this study ranged from 2 to 100 mg mL^–1^. The surface tension values of the prepared BSA concentrations range from 62 to 58 mN m^–1^ and seem to be independent of the concentration for the range 2–20 mg mL^–1^ (Figure S3a). Figure 2a shows the plastron coverage ratio vs immersion time curves of fluoropolymer-coated black Si surfaces in water and five BSA concentrations. Our results indicate an inverse relationship between the BSA protein concentration and plastron stability (Figure 2b). Given that the surface tension does not change significantly across various BSA concentrations, the shorter plastron lifetime at higher BSA concentrations is attributed to the increased protein adsorption onto the black Si surface due to the increased protein content in the bulk solution. Subsequently, protein contamination alters the surface chemistry of the SHB surface, rendering it more hydrophilic. This modification facilitates liquid infiltration into surface trenches, displacing air and diminishing plastron longevity. A similar finding was in a study conducted by Wang et al.^35^ where they conducted a plastron lifetime comparison between different BSA concentrations along with ethanol solutions that have the same surface tension. Their conclusion was that in addition to the influence of surface tension, protein adsorption had a significant role in plastron dissipation. The plastron lifetime of their SHB surface is limited to just 5–10 min at BSA protein concentrations of 0.01, 0.10, and 1.00 mg mL^–1^. In contrast, some of our surfaces exhibit plastron lifetimes ranging from hours to days, even at BSA concentrations as high as 100 mg mL^–1^. Their surface was fabricated by spray-coating a silicon wafer with an SHB coating, resulting in a thin layer of randomly distributed nano/micro-isolated pores.^49^ In comparison, our surfaces feature deeply etched silicon micropillars with an open structure, which sustain a larger plastron volume and a more stable liquid–air interface. This significant improvement highlights the long-term biofluid repellency effect of our surface design.

Leibner et al.^50^ showed that the adsorption of human serum albumin onto the polytetrafluoroethylene SHB surface is minimal when plastron is maintained on the surface. However, when the solution is degassed and the plastron is lost (Wenzel state), the authors observed a positive correlation between protein adsorption on the surface, measured by a radiometric method, and its concentration until reaching a maximum adsorption amount when the whole surface area is saturated with proteins at a concentration of about 45 mg mL^–1^. Huang et al.^51^ have further corroborated that the plastron of the SHB regions, on a patterned superhydrophilic/SHB TiO_2_ nanotube surface, effectively hindered protein adsorption from BSA and FBS solutions. Intriguingly, following plastron removal through sonication, protein adsorption on the SHB regions surpassed that on the superhydrophilic regions. This outcome suggests a more substantial interaction between the protein and the hydrophobic coating compared with the hydrophilic surface. Roach et al.^52^ reported a higher binding affinity of fibrinogen and albumin proteins to hydrophobic surfaces compared to hydrophilic surfaces.

The contact angles of the different BSA protein solutions on the black Si SHB surface are independent of the concentration (see Figure S3b). The receding contact angle for all protein solutions on the black Si SHB surface (∼134°) is dramatically lower than that for water (∼169°) due to the increasing solution adhesion forces to the surface because of the protein adsorption on the hydrophobic coating. On the other hand, the protein adsorption does not affect the advancement of the solution on the surface, making the protein solution advancing contact angle similar to pure water (∼170°). Aldhaleai and Tsai^53^ showed that as cationic surfactant concentration increases, contact angles and plastron durability decrease.

Various biofluids were prepared to investigate the impact of biomolecule identities and concentrations on SHB surface plastron lifetime. These biofluids comprised RPMI 1640 cell medium with distinct compositions including BSA, FBS, glucose, or a combination thereof. Table S2 shows each biofluid abbreviation and its composition. Figure 2c shows the plastron lifetime curves of 10 μm Si pillars (25 μm pitch size and 15 μm pillar height) immersed in the prepared biofluids. The plastron of the micropillared surface immersed in pure water is very stable, lasting over a month. However, as shown in Figure 2d, when immersed in the biofluids, the plastron lifetime dramatically decreases, and its decrease depends on the type and concentration of the added biomolecules. Elevated concentrations of all three additives resulted in a shorter plastron lifetime.

FBS contains mainly proteins (albumin, fibronectin, and globulins), sugars (glucose), growth factors, salts, etc. Studies have shown that fibronectin is problematic for biomedical devices due to its high adsorption affinity on solid surfaces.^54^ The biofluid 10% FBS is widely used in biomedical research for cell culture. It was found that the plastron lifetime of the 10 μm pillared surface immersed in 10% FBS is almost 3 days, indicating that such a surface could potentially be used in applications where a time frame of hours to few days is required.

To assess the substantial contribution of biomolecules, other than albumin, fibronectin, and glucose, in FBS on plastron dissipation, 10% FBS with its equivalent concentrations of glucose (0.125 mg mL^–1^) and total protein (8 mg mL^–1^) utilizing BSA only were compared. The investigation revealed comparable plastron lifetime curves for both solutions (10% FBS and 8BSA+0.125Glu), suggesting that proteins with high adsorption affinity and glucose are the primary components of FBS influencing SHB surface plastron stability. Similarly, a comparison between 100% FBS and 80BSA+1.25Glu yielded similar results. The slightly shorter plastron lifetime of 80BSA+1.25Glu solution may be attributed to the smaller surface tension of this solution compared to 100% FBS (Figure S4). However, when comparing 100% FBS with 50BSA+10% FBS, which have roughly equivalent BSA quantities but differ in total protein content, the observation was a faster plastron dissipation in 100% FBS, highlighting the genuine impact of fibronectin and other proteins such as globulins on plastron stability. The strong impact of glucose on plastron lifetime is evident in the curve of 1.25Glu solution in Figure 2c. A concentration of 1.25 mg mL^–1^ of glucose alone exhibited a greater effect on the plastron compared to 8BSA+0.125Glu and 10% FBS solutions, as shown in Figure 2d. Lv et al.^55^ showed that the superhydrophobicity of an aluminum surface was lost when the glucose concentration has reached 1 mg mL^–1^. The authors mentioned that the contact angle of the solution on the SHB surface was 148.7°, and the surface tension of glucose solution is 50.25 mN m^–1^. Figure S4 shows the surface tension of the studied biofluids and their dynamic contact angles on HL and HB smooth Si reference surfaces, as well as on the SHB 10 μm pillared Si surface. The surface tension of the cell culture medium (RPMI 1640) is slightly smaller than water. The addition of BSA, FBS, or glucose to the cell medium decreases its surface tension from 70 to 54–67 mN m^–1^ (Figure S4a). Increasing the concentration of each biomolecule for certain concentration ranges does not significantly decrease the surface tension further due to the droplet surface saturation with the added biomolecule at this point (Figure S3a). Absolom et al.^56^ reached a constant surface tension of 61 mN m^–1^ for human serum albumin solution with increasing the protein concentration from 0.35 to 5.5 mg mL^–1^. Thi-Yen Le et al.^57^ observed a constant equilibrium surface tension of 51.5 mN m^–1^ for seven concentrations of BSA solutions lower than 0.1 mg mL^–1^. Figure S4b-d shows the dynamic contact angles on HL and HB smooth Si reference surfaces as well as on the SHB 10 μm pillared Si surface. A detailed discussion is found in the Supporting Information (Section S2.2). Although the surface tension and dynamic contact angle values remain similar across biofluids with different compositions, the plastron longevity varies significantly with changes in biofluid composition. This substantiates the pivotal role of biomolecular adsorption onto the SHB surface as the primary contributor to plastron dissipation because of the increasing surface energy of the SHB surface. Furthermore, this suggests that the extent of superhydrophobicity of a surface, as indicated by dynamic contact angles, does not always correlate with its durability.

### Design of Long-Term Biofluid-Repellent Superhydrophobic Surfaces

Next, the effects of the feature size, solid fraction, and plastron film height of micropillared superhydrophobic surfaces are investigated to isolate the impact of each factor and establish optimized design parameters for the long-lasting biofluid-repellent effect of SHB surfaces. Table 1 presents the diverse parameters studied. The pillar height is varied to investigate the effect of air film volume on plastron lifetime. The pillar size and spacing are varied to study the effect of the Cassie fraction and the feature size of the pillars on the plastron stability.

**Table 1:** Surfaces Used to Study the Effect of Superhydrophobic Surface Parameters on Plastron Stability in Biofluids

studied parameter	pillar Size [μm]	solid fraction [%]	pitch [μm]	pillar height [μm]
pillar size	5	19.6	10	30
	10^a^	19.6	20	30
	20	19.6	40	30
	30	19.6	60	30
	40	19.6	80	30
	50	19.6	100	30
solid fraction	10^a^	22.7	20	15
	10	14.5	25	15
	10	7.4	35	15
	10	2.5	60	15
pillar height	10	22.7	20	15
				40
	10	14.5	25	15
				40
	10	7.4	35	15
				40
	10	2.5	60	15
				40

a 10 μm pillars with 20 μm pitch size has two different solid fractions because of different lattice structures: hexagonal (22.7%) and orthogonal lattice (19.6%).

#### Pillar Size

**Figure 3:**
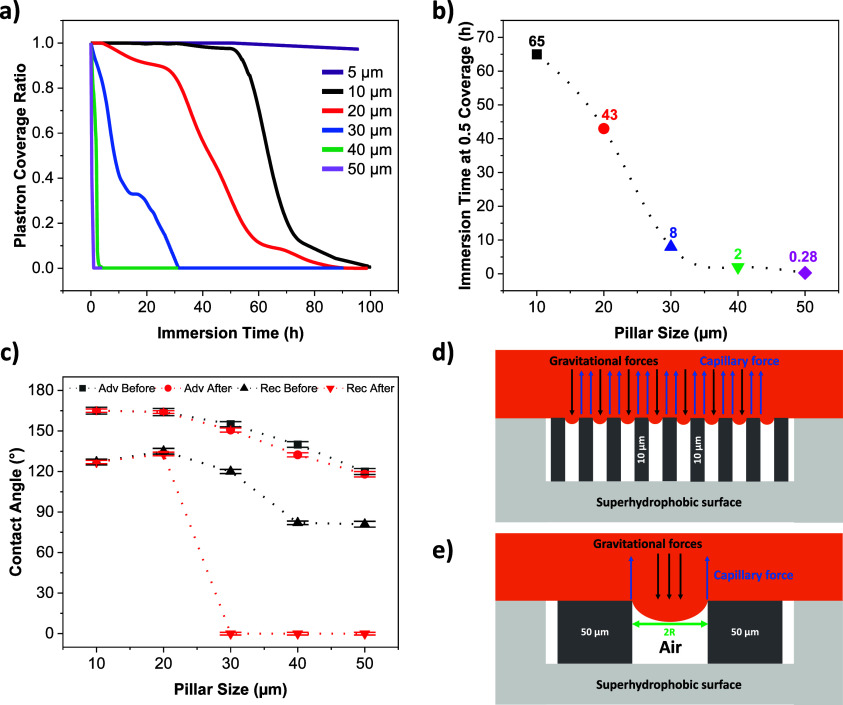
Effect of pillar size on plastron lifetime in biofluids. (a) Plastron coverage ratio of 9 cm^2^ Si micropillared surfaces with different pillar sizes (5–50 μm) vs the immersion time in RPMI medium containing 80 mg mL^–1^ BSA and 2 mg mL^–1^ glucose. (b) Effect of pillar size on plastron durability measured at 50% plastron area coverage. (c) Water advancing (Adv) and receding (Rec) contact angles of the five surfaces before and after immersion in the biofluid for a week. Schematic illustration of the local force balance of downward gravitational forces and upward capillary forces in a droplet curvature between adjacent (d) 10 μm and (e) 50 μm micropillars. *R* is the curvature tip radius (half of the pillar spacing).

The impact of micropillar scale variation was studied by altering both the pillar size and pitch by 10-fold, ranging from 5 and 10 μm to 50 and 100 μm, respectively. Six pillar sizes were studied: 5, 10, 20, 30, 40, and 50 μm. The volume of the plastron film remains identical across all six surfaces (same Cassie solid fraction and height), eliminating air diffusion route discrepancies. Instead, the variation in the air pushing bubble route predominantly hinges on the pillar size and spacing, making it the primary pathway for plastron dissipation variations in these pillared surfaces. The six surfaces were fully submerged in RPMI medium supplemented with 80 mg mL^–1^ BSA and 2 mg mL^–1^ glucose, and the results are shown in Figure 3a,b. A distinct effect emerged: the smaller the pillars, the longer the plastron lifetime. Since the solid fraction is constant across all surfaces, the increased spacing between larger pillars accelerates the transition from the Cassie to the Wenzel state.

The pillar size 50 μm has a relatively short plastron lifetime (∼1 h), while the pillar size 10 μm has a plastron lifetime of about 100 h. This is due to two main factors: varying the wettability behavior and disturbing the localized force balance. Figure 3c shows the surfaces’ water wettability before and after being immersed in the biofluid for a week. Focusing on the measurements before biofluid immersion, a noticeable trend is observed: there is a distinct reduction in water dynamic contact angles as the pillar size surpasses 20 μm, signaling a decline in superhydrophobicity. The receding contact angles of pillar sizes of 40 and 50 μm were around 80°, which is a similar value for the reference flat hydrophobic surface, suggesting a Wenzel wetting state upon droplet receding. Looking at the surfaces’ water wettability after the immersion in the biofluid for a week, a clear distinction is noticed: 10 and 20 μm pillar sizes maintained their hydrophobic properties, while the larger pillars displayed increased post immersion wetness, as they exhibited a Wenzel transition and strong pinning effect with a 0° receding angle, indicating a changed surface chemistry to hydrophilic as biomolecules are adsorbed on the hydrophobic coating. In this context, both superhydrophobicity and the plastron lifetime exhibit an increase as the pillar size decreases. This implies that superhydrophobicity, in that specific case, reinforces plastron stability. Figure 3d,e shows the local force balance between the upward capillary forces of the biofluid, driven by surface tension, and the downward gravitational forces (hydrostatic pressure) of the fluid. Rathgen et al.^58^ noted that an SHB surface undergoes a Cassie to Wenzel state transition when the water–air interface experiences a dynamic pressure exceeding a specific threshold called the critical pressure (P_C_). According to the Young–Laplace equation:^33^
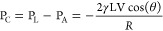
3where P_L_ and P_A_ are the pressures of the liquid and air respectively, γ_LV_ is the liquid surface tension, θ is the liquid contact angle with the planar surface, and *R* is the capillary radius and is directly related to the pillar spacing. In the case of bigger pillars, the greater pillar spacing (and *R* value) decreases the critical pressure (P_C_) at which the pillar is lost. Simultaneously, capillary forces decrease due to a reduced three-phase contact line. Additionally, when dealing with a biofluid with lower surface tension than water, liquid pressure resistance is also diminished. As *R* goes to the nm scale, such as in black Si, the critical pressure of the wetting transition increases greatly. This eliminates the bubble mechanism route of plastron dissipation in black Si and air diffusion, becoming the solo mechanism of dissipation in the case of pure water. However, in biofluids, biomolecular adsorption significantly alters the surface chemistry (decreasing θ), thereby accelerating the plastron loss. Koc et al.^59^ observed that BSA protein adsorption, on an SHB surface, was greater for higher scale roughness (4 μm, 800 nm) compared to smaller roughness features (10 nm) that adsorb much less. This is clearly due to higher solid–liquid interface area in the microscale roughness, which increases the protein adsorption probability and affinity. In cases where circular pillars are arranged in hexagonal or square arrays (such as in our study), the effective capillary radius *R*_eff_ can be calculated using the following equation:^33^
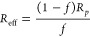
4where *f* is the solid fraction and *R*_p_ is the pillar radius. This relationship indicates that smaller pillar sizes result in a reduced capillary radius, enhancing resistance to high pressures and increasing plastron durability.

#### Solid Fraction

**Figure 4:**
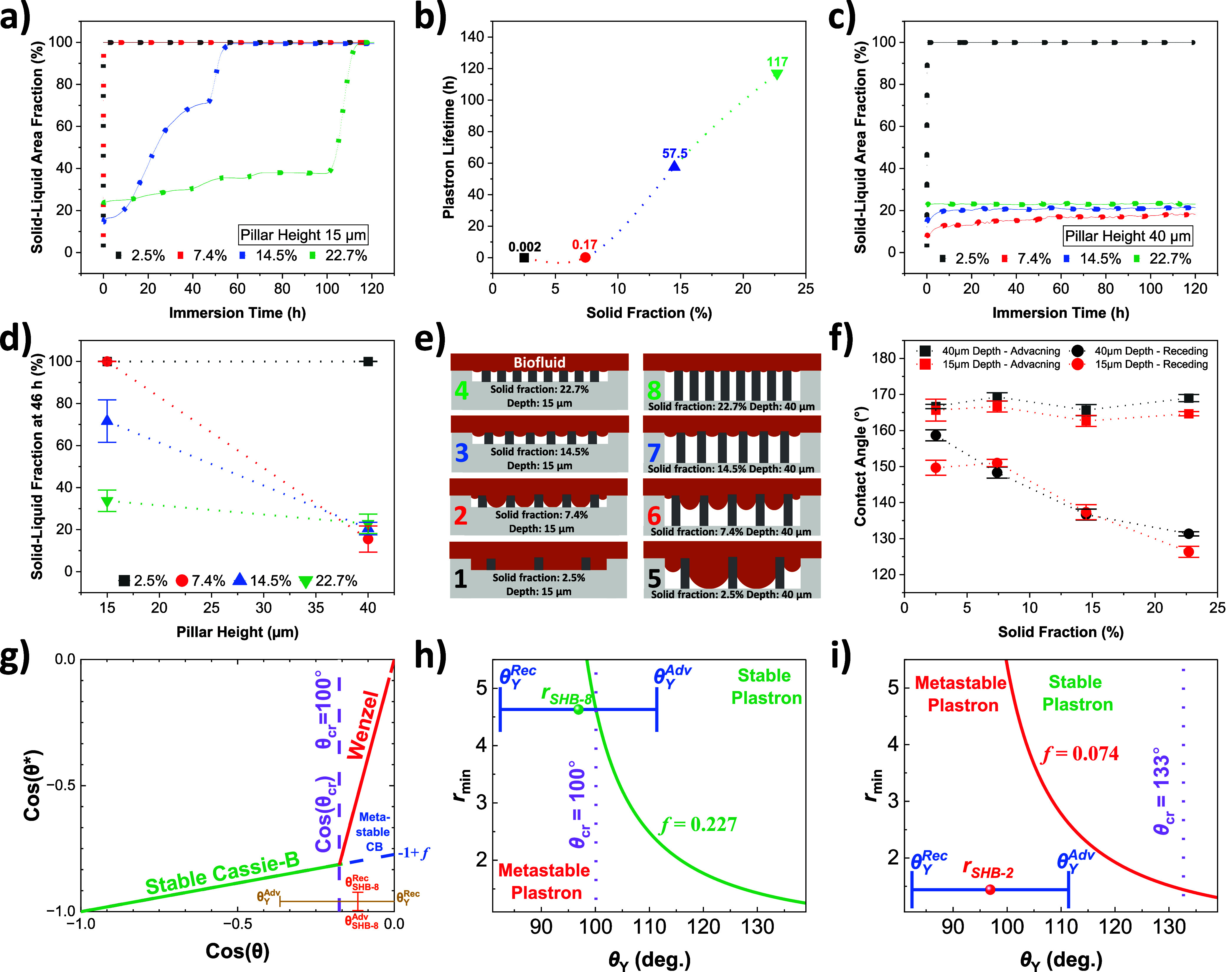
Effect of the Cassie solid fraction (*f*_*o*_) and pillar height on the plastron lifetime. The solid–liquid area fraction (*f*) of the 10 μm pillared Si surface vs immersion time in RPMI medium containing 50 mg mL^–1^ BSA and 10% FBS. Surfaces (a) with 15 μm pillar height and (c) with 40 μm pillar height, both with four different solid fractions. Effect of the (b) surface solid fraction and (d) pillar height on the plastron lifetime. (e) Schematic illustration of the wetting state of the eight studied surfaces when immersed in a biofluid along with each surface number. (f) Water dynamic contact angles of the eight micropillared SHB surfaces. (g) Lafuma–Quéré diagram: Cassie or Wenzel contact angles (cos(θ*)) as a function of Young's contact angle (cos(θ)), based on the solid–liquid area fraction of the SHB-8 pillared surface. Marmur’s diagram: the minimum roughness (*r*_min_) as a function of Young's contact angle based of the roughness and solid–liquid area fraction of surfaces (h) SHB-8 and (i) SHB-2.

Next, the solid fraction of the micropillared surface is altered while keeping the pillar size and height fixed. Figure 4a shows the solid–liquid area fraction (*f*) of 10 μm Si pillared SHB surfaces with different starting Cassie solid fractions (*f*_*o*_) immersed in RPMI 1640 medium supplemented with 50 mg mL^–1^ BSA and 10% FBS. A stable plastron would be interpreted if the solid–liquid area fraction (*f*) remains as close as possible to its initial value (*f*_*o*_). The pitch sizes of the four surfaces are 60, 35, 25, and 20 μm for 2.5, 7.4, 14.5, and 22.7% solid fractions, respectively. As shown in Figure 4b, the lowest solid fraction (2.5%) has an immediate Cassie to Wenzel state transition (100% solid–liquid fraction) as soon as it is immersed in the biofluid, while the plastron of 22.7% Cassie solid fraction surface took around 116 h to totally dissipate. As the Cassie solid fraction decreases, *R*_eff_ increases (eq 4), decreasing the critical pressure (Pc) in eq 3. This reduces the liquid–air interface stability at a certain liquid pressure, increasing its curvature further. If the curvature becomes large enough to reach the bottom of the trenches, it accelerates the Cassie to Wenzel state transition. The surface with a 2.5% Cassie solid fraction has the largest air film volume. Nonetheless, it is the fastest to reach the Wenzel state, confirming that the major plastron dissipation route for the micropillared surfaces is the air-pushing bubble mechanism and not air diffusion.

When designing an SHB surface for biomedical applications, the solid fraction–plastron stability relationship shown in Figure 4b highlights the need to identify a solid fraction large enough to effectively stabilize the Cassie state and withstand biofluid hydrostatic pressure for the desired duration. Simultaneously, it is important to note that increasing the solid fraction beyond this point will only amplify biomolecular adsorption, presenting a disadvantage for this surface. For instance, the plastron stability of 100 nm Si pillars with black Si with the same surface coating was compared. Although the solid fraction of the nanopillars is larger than the black Si, it was observed that the plastron of the black Si plastron is more stable under the used biofluid (Figure S5). This can be attributed primarily to the larger tips of the nanopillars (100 nm) in comparison to the much smaller tips of the black Si (5–10 nm). Additionally, the substantial increase in the solid–liquid interface area enhances the likelihood of protein adsorption without contributing to greater liquid–air stability.

#### Pillar Height

The pillar height effect on the plastron lifetime was studied by fixing the pillar size for four sets of solid fractions. Figure 4a and Figure 4c show the solid–liquid area fraction over time of the micropillared Si surfaces with 15 and 40 μm pillar heights, respectively. Figure 4d shows the effect of pillar height on the plastron stability after 46 h of immersion in RPMI 1640 medium supplemented with 50 mg mL^–^^1^ BSA and 10% FBS. Stable plastron is indicated by the solid–liquid area fraction (*f*) remaining as close as possible to its initial value (*f*_o_). The effect of pillar height seems to greatly depend on the Cassie solid fraction. For the highest solid fraction (22.7%), increasing the pillar height only slightly enhanced plastron stability. On the other hand, for the lowest solid fraction (2.5%), increasing the pillar height did not increase the plastron lifetime at all. For the two solid fractions in between (7.4 and 14.5%), the plastron stability is enhanced significantly and especially for 7.4%.

For the 7.4% solid fraction, the 15 μm pillar height is lower than its pillar spacing (25 μm), leading the concave meniscus of the biofluid to be able to reach the bottom of the trenches at the current hydrostatic pressure, causing a fast Cassie to Wenzel state transition (Figure 4e, 1–4). Meanwhile, for the 40 μm pillar height sample, the height is larger than the pillar spacing (25 μm), which does not allow the concave meniscus to reach the trench bottom, giving a stabler Cassie state (Figure 4e, 5–8). The two pillar heights with the 2.5% solid fraction showed similar plastron stability because the 15 and 40 μm pillar heights are both smaller than the pillar spacing (50 μm) of this solid fraction. For the higher solid fractions (14.5 and 22.7%), the plastron is generally more stable with bigger heights due to an increased air film volume.^60^ Since the Cassie state is stable for both heights, air diffusion is an important route of plastron dissipation. The surface with the 22.7% solid fraction and 40 μm pillar height kept its Cassie state and plastron for more than several weeks. A video was taken of a sample surface plastron film rupturing after it was withdrawn from the biofluid, and the surface was left dry (see Supporting Video 2). Meanwhile, in the case of the 2.5% solid fraction surface, the Wenzel state was characterized by a fully wet surface upon surface withdrawal.

To compare the two heights of the 22.7% solid fraction further, the 15 and 40 μm heights samples were immersed in a biofluid of 100 mg mL^–1^ BSA and 2 mg mL^–1^ glucose dissolved in FBS. Figure S6 shows the plastron lifetime curves of both surfaces. Notably, an approximately 3-fold increase in the plastron lifetime was detected for the sample with a 40 μm height (30 h) compared to the one with a 15 μm height (10 h). This increase is directly proportional to the air volume increase in the two samples. More discussion is found in the Supporting Information (Section S2.3).

A Cassie state is attained in most of the studied surfaces as confirmed by plastron silver-like reflected color detected by our setup, contact angle measurement (Figure 4f), and the applicability of the following equation:^12^
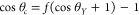
5where θ_c_ is the Cassie’s contact angle (measured on the SHB surface), *f* is the solid fraction, and θ_Y_ is the Young’s contact angle (measured on a flat HB reference surface) (see Section S2.4, Supporting Information). Remarkably, in this case, superhydrophobicity does not appear to enhance plastron stability, as observed with pillar size. Although there is no distinct difference in superhydrophobicity between the two heights, a clear trend is observed with the solid fraction: as the solid fraction increases, superhydrophobicity decreases, while plastron stability increases. This underscores the limitation of contact angle measurements in predicting plastron stability in liquid immersion scenarios, given their reliance on small droplets with significantly lower hydrostatic pressure compared to the conditions faced by the surface’s plastron when fully submerged in a liquid. Therefore, there is a need for a new characterization approach to measure the stability of the SHB surface submerged in a fluid. The critical contact angle (θ_cr_) for a Cassie to Wenzel state transition can be calculated using the Lafuma and Quéré equation:^61^
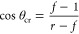
6where *f* is the solid fraction in the Cassie state and *r* is the surface roughness ratio. When the Young’s contact angle of the smooth surface is greater than θ_cr_, the wetting state is expected to be a Cassie state with stable plastron. Meanwhile, if this contact angle is smaller than θ_cr_, a Wenzel state is the wetting state. The stability of the plastron can also be analyzed in terms of surface roughness *r*. Marmur showed that there is a given minimum roughness (*r*_min_) at which the superhydrophobicity can be stable underwater at a certain solid fraction (*f*) and Young's contact angle (θ_Y_) (*i.e*., contact angle on the smooth HB ref. surface).^8^ Marmur derived the following equation:^8^
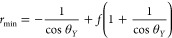
7

Based on Lafuma and Quéré (eq 6)^61^ and Marmur (eq 7) diagrams,^8^ Tesler et al.^20^ suggested that plastron stability can be characterized by assessing three parameters: (i) the dimensionless Wenzel roughness parameter (*r*), (ii) the solid–liquid area fraction (*f*), and (iii) Young’s contact angle (θ_Y_). Figure 4g–i shows the Lafuma–Quéré critical contact angle and Marmur’s minimum roughness diagrams for SHB surfaces number 8 and 2 (Figure 4e). While SHB-8 has its Young-HB reference advancing angle in the stable Cassie region and higher than θ_cr_, the SHB-2 surface contact angle range (Rec. to Adv.) is entirely out of the stable Cassie region and lower than θ_cr_. This perfectly aligns with our experimental findings demonstrated in Figure 4a–d. SHB-2 experiences an immediate collapse of the liquid–air interface through the sag transition mechanism (pillar height < sag), whereas SHB-8 undergoes a slower depinning collapse.^62^ More discussion on this section can be found in the Supporting Information (Section S2.5).

#### Effect of Surface Chemistry

**Figure 5:**
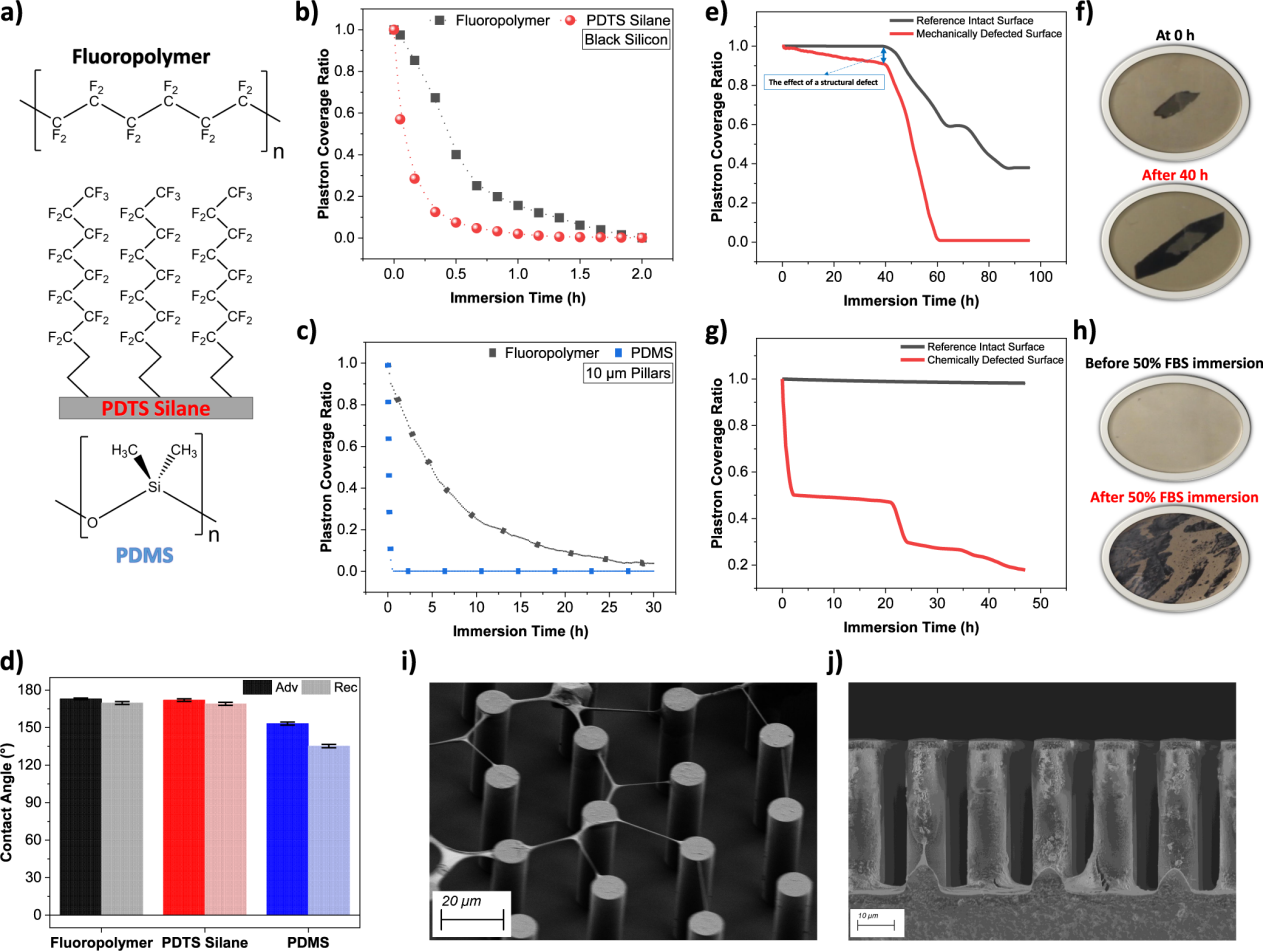
Effect of surface chemistry and defects on plastron stability in biofluids. (a) Chemical structure of the fluoropolymer (top), PDTS self-assembled monolayer (middle), and PDMS (bottom). Plastron coverage ratio over immersion time of (b) black Si coated with the fluoropolymer and PDTS silane and (c) Si 10 μm pillars coated with the fluoropolymer and PDMS 10 μm pillars, immersed in FBS serum supplemented with 100 mg mL^–1^ BSA and 2 mg mL^–1^ glucose. (d) Water dynamic contact angles of the three surface chemistries. The effect of mechanical and chemical defects on plastron stability is also studied. (e) Plastron coverage ratio of intact reference and mechanically defective 10 μm pillared surfaces over immersion time in RPMI medium supplemented with 80 mg mL^–1^ BSA and 2 mg mL^–1^ glucose. (f) Optical images of the plastron around the structural defect at the beginning and after 40 h* of immersion. (g) Plastron coverage ratio of intact reference and chemically defective 10 μm pillared surfaces over time underwater. The chemical defects were made by immersing the surface in 50% FBS for 3 days. (h) Optical images of the chemically defective surface plastron in water before and after FBS immersion. (i) SEM image of the pillars that kept a Cassie state during their immersion in a biofluid. The image shows biodeposited materials only on the pillar top level. (j) SEM image of the pillars that transitioned to the Wenzel wetting state during their immersion in a biofluid. The image shows a deposited biofilm all over the pillars. *The spreading of Wenzel state formed a hexagonal shape around the defect due to the hexagonal array lattice of the pillars.

A small test was conducted by comparing three different surface chemistries to each other (Figure 5a). Two fluorinated coatings (fluoropolymer and fluorinated self-assembled monolayer silane) and a PDMS surface contain methyl groups. Figure 5b,c shows the plastron lifetime of these three coatings when immersed in 100 mg mL^–1^ BSA and 2 mg mL^–1^ glucose dissolved in FBS. Among these coatings, the fluoropolymer coating on the 10 μm silicon pillars exhibits the longest plastron lifetime. Despite the PDTS silane SAM coating being fluorinated, it demonstrates a shorter plastron lifetime compared to the fluoropolymer coating on the black Si surface. The methyl-ended surface chemistry on the PDMS pillars results in the shortest plastron lifetime, while the fluoropolymer surface chemistry on the 10 μm silicon pillars significantly extends the plastron lifetime. Nonetheless, it is important to note that the mechanical stability of PDMS in the presence of biofluid also contributes significantly to its shorter plastron lifetime. Figure S7 shows the PDMS pillars sticking to each other after their short immersion in the biofluid. Roach et al.^63^ reported that the affinity of fibrinogen and albumin is higher for CH_3_-terminated coatings compared to hydrophilic coatings. This increases the adsorption of the proteins on the solid surfaces, enabling a Cassie to Wenzel transition. Figure 5d shows the effect of a surface coating on water contact angles. Figure S8 shows the plastron lifetime of the three coatings on the SHB surface underwater.

Koc et al.^59^ observed a close extent of protein adsorption on methylated flat and nanoneedle (10 nm) SHB surfaces compared to their fluorinated correspondents. However, for SHB surfaces with 800 nm and 4 μm structured features, the fluorinated surfaces had an almost 2-fold decrease in protein adsorption compared to the methylated surfaces. This shows that overall, fluorinated surface chemistry is more resistant to protein adsorption.

#### Effect of Mechanical and Chemical Defects

When the air-pushing bubble mechanism is the predominant route of plastron dissipation, it was observed that the collapse of the Cassie state into the Wenzel state at one point on the surface would assist the transition of neighboring sites into the Wenzel state. Mechanical defects such as missing or destroyed pillars mean that at this site, the pillar spacing is larger than the rest of the surface, which leads to a bigger curvature of the liquid–air interface at this site. Chemical defects such as deposited proteins on the surface would change the surface chemistry from hydrophobic to hydrophilic at a site, leading to faster plastron dissipation. Therefore, the effect of mechanical and chemical defects on the plastron lifetime of the Si micropillared surface is studied.

A mechanical defect was made by scratching a 10 μm pillared surface with metal tweezers, followed by subsequent cleaning. The defective surface and an intact reference surface were then immersed in RPMI medium containing 80 mg mL^–1^ BSA and 2 mg mL^–1^ glucose. The graph in Figure 5e shows a significant plastron loss for both surfaces after 40 h. However, the defective surface lost its plastron completely, whereas the reference surface maintained it for an additional 60 h at least. Figure 5f shows an image of the defect at 0 and 40 h. Notably, the defects served as the first sites to transition into the Wenzel state, while the reference surface retained its plastron within the first 40 h. Mechanical defects serve as hydrophilic nucleation sites for liquid infiltration, accelerating the Cassie-to-Wenzel transition by disrupting local capillary forces. Domingues et al.^64^ demonstrated that in the case of single, isolated mushroom-like pillars, localized physical damage (e.g., broken pillars) leads to rapid wetting at the defect site. This discontinuity enables the liquid to displace the trapped air and spread into undamaged regions, ultimately resulting in a complete surface wetting. To counter this issue, they proposed the use of mushroom-like or doubly re-entrant cavities, where the solid structure remains continuous and air pockets are isolated, thus enhancing resistance to wetting even in the presence of defects.

Additionally, the chemical defect effect was studied by using a 10 μm pillared surface that was immersed in 50% FBS for 3 days. Many biomolecules, especially proteins, will be adsorbed on pillar tops in the areas that were in the Cassie state and on the entire pillar surface and trenches of the areas that were in the Wenzel state. This led to changing the surface chemistry of many areas of the surface to more hydrophilic. This leads to different wettability behavior, as shown in Figure 3c, where the contact angle comparison before and after immersion in the biofluid suggested a change in the surface chemistry by becoming more hydrophilic. Figure 5g shows a comparison of the plastron lifetime of the surface when immersed in water before and after 50% FBS immersion. An immediate loss of a significant percentage of the plastron happened on the defective surface when immersed in water, especially in the areas that were in the Wenzel state in the 50% FBS. Figure 5h shows the Wenzel state areas formed by the chemical defects caused by protein adsorption. Figure 5i and Figure 5j show the SEM images of the Si pillars demonstrating protein deposition in the Cassie and Wenzel state, respectively. It is evident that in the Wenzel state, protein adsorption is significantly amplified compared to a flat surface, attributable to the larger solid–liquid area characteristic of this wetting state (roughness >1). Figure S9 shows the SEM images of deposited biofilms on the SHB surface after their immersion in a biofluid. Moulinet and Bartolo investigated the impact of missing pillars and chemically defective pillars on the collapse pattern of the liquid–air interface during sessile droplet evaporation. They observed similar results to our findings on submerged SHB surfaces.^65^

## Conclusions

Extensive research has focused on superhydrophobic surfaces for their water-repellent properties, holding promise across various applications, including the biomedical field. Despite this, a comprehensive understanding of their efficacy and longevity as biofluid-repellent materials has been lacking. This study systematically investigated the plastron stability of superhydrophobic surfaces fully immersed in various biofluids using an optical monitoring setup. Our work establishes a critical advancement in the field by demonstrating that plastron stability exceeding 120 h can be achieved in complex biofluids containing high concentrations of proteins and glucose. This is a significant improvement over previously reported lifetimes of minutes to hours, enabled by the optimized interplay of the surface morphology and chemistry. By bridging the gap between fundamental water repellency and practical biofluid repellency, our study sets the stage for the development of long-lasting biomedical superhydrophobic surfaces.

Plastron dissipation pathways were observed to be impacted by both surface texture and the biofluid, with the predominant plastron dissipation mechanisms identified as air diffusion and the air-pushing bubble mechanism. Generally, plastron lifetimes are shortened in biofluids compared to pure water, primarily attributed to the adsorption of proteins (albumin and fibronectin) and glucose onto the solid–liquid interface through hydrophobic–hydrophobic interactions and second due to the lower surface tension of biofluids. Faster plastron dissipation was observed in biofluids with a higher biomolecule concentration and lower surface tension.

Our findings indicate several useful design criteria for superhydrophobic surfaces when longer plastron lifetimes are required in biofluid immersion. First, downscaling the pitch and the pillar size is beneficial if at the same time the height of the plastron is not altered. Second, increasing the height of the plastron can be very beneficial, especially for low solid fraction surfaces. Third, there exists a trade-off in the solid fraction where a higher solid fraction increases the plastron lifetime but at the same time reduces the overall repellency of the surface. Overall, the data show that superhydrophobic surfaces with proper design might well be able to sustain plastrons of complex biofluids for at least days, which will potentially open them for biomedical applications. Some suggested future works to build on this study are as follows: there is a need to provide a new characterization approach to determine the superhydrophobicity under fluids since the contact angle measurement does not always align with plastron stability results; further studying the effect of surface chemistry on the plastron lifetime in biofluids to find the best biofluid-repellent surface coating; testing the long-term stability of superhydrophobic surfaces in more complex biofluids such as cell cultures and blood; theoretical studies to validate the findings of this study.

## Supporting Information Available

Detailed experimental methods of fabrication protocols for superhydrophobic surfaces; additional discussions on plastron stability, dynamic contact angle measurements, mechanical robustness, and Marmur minimum roughness; supplementary tables of surface geometry parameters and biofluid composition and abbreviations; nine figures of the SEM images of surface textures and deposited biofilm, surface tensions and dynamic contact angles of biofluids, plastron stability, and coverage ratio of different surfaces under various conditions (PDF)
Supporting Video 1: bubble mechanism during plastron dissipation on a Si 10 μm pillared superhydrophobic surface over 120 h (MP4)
Supporting Video 2: air film rupture of a superhydrophobic surface after 1 week of immersion in a complex biofluid (MP4)
