# Supplementary material for: Stable Air Plastron Prolongs Biofluid Repellency of Submerged Superhydrophobic Surfaces
Source: Langmuir. 2025 Jan 15;41(3):1807–20. doi: 10.1021/acs.langmuir.4c04259 (PMC11780736; doi:10.1021/acs.langmuir.4c04259)
Supplement: Supplementary file 1 — la4c04259_si_001.pdf [file la4c04259_si_001.pdf]

Supporting Information

Stable Air Plastron Prolongs Biofluid Repellency of Submerged Superhydrophobic Surfaces

Mohammad Awashra, Seyed Mehran Mirmohammadi, Lingju Meng, Sami Franssila, and Ville Jokinen**

Aalto University

School of Chemical Engineering

Department of Chemistry and Materials Science

Tietotie 3, Espoo 02150, Finland

E-mails : mohammad.awashra@aalto.fi, ville.p.jokinen@aalto.fi

Table of Contents

1. Additional Experimental Methods S2
1.1. Superhydrophobic Surfaces Fabrication S2
2. Additional Discussion S6
2.1. Effect of Biofluids on Nano- and Micro-Plastron Stability S6
2.2. Dynamic Contact Angles of the Studied Biofluids S8
2.3. Pillar Height vs. Mechanical Robustness S10
2.4. The Applicability of Cassie-Baxter Equation S10
2.5. Marmur Minimum Roughness S12
REFERENCES S13
3. Supplementary Tables S14
4. Supplementary Figures S15
5. Supporting Videos Description S24

1. Additional Experimental Methods

1.1. Superhydrophobic Surfaces Fabrication

Using standard optical lithography, the Si micropillared surfaces were fabricated by first applying an adhesive layer of hexamethyldisilizane (HMDS) (Vapor Prime Oven, YES-3). Then, photolithography was performed as the following: AZ 5214E photoresist (MicroChemicals) was spin coated on the wafer (4000 rpm for 30 s), soft baked (90 °C, 2 min), exposed for 3 s with different photomasks (Süss MicroTec MA-6 with 365 nm wavelength), and developed for 1 min in AZ 351B (Merck). Next, the unprotected areas were etched using BOSCH deep reactive ion etching (DRIE) process for different number of cycles to achieve the required heights. The process parameters (Oxford PlasmaPro 100 Estrelas ICP-DRIE) were 20 °C temperature, 50 and 40 mTorr pressure, ICP power 1800 and 1500 W, and the gas flows were 10 and 200 sccm for O_2 and 300 and 10 sccm for SF_6 for step one and two of each cycle, respectively. The photoresist was then removed by ultrasonication in acetone. The wafer was then covered with a fluoropolymer hydrophobic coating using a PECVD process (Oxford Plasmalab 80Plus). The process parameters were 250 mTorr pressure, 50 W power, and 100 sccm CHF_3 flow.

The black Si SHB surface was fabricated using a maskless cryogenic deep reactive ion etching (DRIE) process on a 4” Si wafer. The process parameters

(Oxford PlasmaPro 100 Estrelas ICP-DRIE) were −125 °C temperature, 5 mTorr pressure, ICP power 1500 W, forward power 10 W, and the gas flows were 15 sccm for O_2 and 30 sccm for SF_6. The wafer was then covered with a fluoropolymer hydrophobic coating as described earlier for Si micropillars.

The Si nanopillared surface was fabricated using electron beam lithography as the following: The Si nanopillar array was fabricated on a 4'' Si wafer. At first, a layer of AR-P 6200.13 (CSAR 62, Allresist GmbH, Strausberg, Germany) was spin-coated onto the wafer by a Laurell WS-400-6NPP spin coater (Laurell Technologies, Lansdale, USA). The spinning process was conducted in a two-stage manner (10 s of 500-rpm spreading and 40 s of 4500-rpm spinning), and then a one-minute soft-baking (150 °C) on a hotplate followed. The electron beam exposure was performed on a Vistec EBPG 5000+ electron-beam lithography system (Raith GmbH, Dortmund, Germany) in the direct-writing mode. During the exposure, beam shot step size was set as 350 nm to isolate each beam shot and get the wanted circle array. The beam current was set to 160 nA and the dose was 90 $\mu C\ cm^{-2}$. After exposure, the wafer was developed in Developer AR 600-546 (Allresist GmbH, Strausberg, Germany) for two minutes with agitation. Consecutive IPA and DI-water rinses were performed right after the development to cease the reaction and clean the surface. The etching mask was created by the lift-off route. A layer of 20 nm AlO_x was deposited onto the resist pattern by electron beam evaporation (IM-9912 evaporator, Instrumentti Mattila,

Mynämäki, Finland). Excessive resist and AlO_x were removed by ultrasonicating in a USC THD/HF ultrasonicator (VWR, Radnor, USA) for 15 minutes at 60 °C in a Remover AR 600-71 (Allresist GmbH, Strausberg, Germany) bath. Three other baths in acetone, IPA, and DI-water were then performed to clean the surface. The surface was then etched using cryogenic deep reactive ion etching (DRIE) process as mentioned before. The nanopillars were then covered with a fluoropolymer hydrophobic coating using the same process used for Si micropillars.

The Cu-PDMS composite surface was fabricated by a facile and low-cost method based on polymer replication. The fabrication process started with Al wet chemical etching in a two-step process; first, Al substrate was etched in a phosphoric acid-based solution for a couple of minutes to remove surface oxide (pre-etching), followed by etching in HCl to obtain rough structure with microscale protrusions. Then, Cu electroless plating was performed to replicate hierarchical micro- and nanostructures from the etched Al into Cu. After deposition of 30 µm thick Cu film, PDMS with the ratio of 10:1 (monomer to the crosslinking agent) was poured on the Cu film. The Al substrate was sacrificially etched away in HCl. The resulting Cu-PDMS composite material was cured at 65 °C for 2 h. The PDMS micropillars were prepared by mixing the monomer and crosslinking agent at a ratio of 10:1. The degassed mixture was poured over a Si wafer master mold having 10 µm holes and then baked at 65 °C for 3 h to solidify.

The PDMS pillars were then peeled off the mold. Finally, a hydrophobic (HB) flat reference surface was made by coating a 4” Si wafer with the fluoropolymer hydrophobic coating and a hydrophilic (HL) flat reference surface was made by cleaning a 4” Si wafer with oxygen plasma. All surfaces were characterized by optical microscopy and scanning electron microscopy (SEM). When a biofilm was imaged, a 10 nm thin gold film was first deposited on the surface to improve SEM imaging.

2. Additional Discussion

2.1. Effect of Biofluids on Nano- and Micro-Plastron Stability

The texture of the surface plays a crucial role in determining the plastron dissipation mechanism. At the nanoscale roughness, in the case of black Si, the collapsing of Cassie state at individual needles is unlikely due to very low liquid-air interface curvature values (almost straight interface). Because of that, Cassie to Wenzel state transition has region- and defect-independent attributes. It is known that the stability of liquid-air interface at the nanoscale roughness is significantly greater when compared to the microscale roughness (see **Eq. 3** in the coming sections). When black Si was immersed in pure water, we observed a plastron lifetime longer than several months. However, in **Figure 1g**, a protein solution is used. Therefore, protein adsorption and air diffusion are the main causes of this faster nano-plastron dissipation on black Si compared to the micro-plastron on the micropillared surface. Consequently, the plastron coverage is decreasing all over the surface simultaneously and a smooth (step-free) plastron coverage ratio vs. immersion time curve is observed. On the other hand, at microscale roughness like in the Si micropillars, the bubble mechanism is more likely to be the plastron dissipation mechanism. Where the pillar size and spacing are large enough for the hydrostatic pressure and gravitational forces of the liquid to compete with its surface tension and capillary forces. The Wenzel state subsequently spreads from any location where the plastron has been lost.

Therefore, unlike nano-structured surfaces, Cassie to Wenzel state transition is region- and defect-dependent. The coverage area of plastron in micropillars surfaces is therefore decreasing heterogeneously in different areas and the air is being pushed to form air bubbles (see **Supp. Video 1**) and the plastron coverage ratio vs. immersion time curve can have steps like shape (as will be shown later) due to air dissipation at largely different rates throughout the experiment.

2.2. Dynamic Contact Angles of the Studied Biofluids

Surprisingly, all biofluids displayed advancing contact angles higher than water and RPMI 1640 on the HL reference surface, despite having lower surface tension than water (**Figure S4b**). This is likely attributed to the heightened hydrophobicity of the droplet surface, resulting from the inclusion of proteins that contain hydrophobic regions, thereby making droplet advancement slightly less favorable, increasing the advancing contact angle. The biofluids have slightly higher advancing contact angles on HB reference and SHB micropillared surface compared to water (**Figure S4c and d**). This could be due to the biofluid's lower wetting affinity for the fluoropolymer coating. Additionally, on SHB surfaces, this behavior may result from the water pinning effect, especially in the presence of surfactants such as proteins. Mohammadi *et al.*[1] found that adding surfactants dramatically alters water contact angles. Comparing contact angles of surfactant solutions to pure liquids with equivalent surface tensions revealed higher contact angles in surfactant solutions. Their study proposed an adsorption hypothesis, suggesting that surfactant adsorption momentarily impedes solution penetration into SHB surface pores and causes a pinning effect. Accardo *et al.*[2] also observed a water pinning effect on a pillared SHB surface when a protein solution was used in a droplet evaporation experiment. On the other hand, the receding contact angle of all biofluids (except RPMI 1640) on the HL and HB reference surfaces was 0-degrees (did not recede from the surface). Moreover, the receding contact angles

on the micropillared SHB surface had a substantial decrease from 137° for water to 114° for all biofluids (**Figure S4d**). Once the droplet encompasses any of the three surfaces, the biomolecules strongly adhere onto the surface, inhibiting droplet recession from the surface (strong pinning effect). Choi *et al*.[3] studied the evaporation of a protein containing droplet on a nanostructured SHB surfaces and observed a strong pinning effect with a receding contact angle of 0-degrees for 10% FBS. They suggested that protein adsorption onto the surface stimulated a Wenzel wetting state transition.

2.3. Pillar Height vs. Mechanical Robustness

These findings indicate that a greater pillar height results in a longer plastron lifetime in a biofluid as long as the pillar height is greater than pillar spacing. However, it is noteworthy that while increasing the pillar height enhances plastron longevity, it concurrently diminishes the surface mechanical robustness, elevating the risk of pillar damage. Among these eight sets of micropillars, a good compromise for an application requiring sustained biofluid repellency for a day could be the surface with 7.4% solid fraction and 40 μm height. This surface is stable enough to maintain the Cassie state under the given hydrostatic pressure, and it features the lowest possible solid fraction, thereby minimizing biomolecule adsorption.

2.4. The Applicability of Cassie-Baxter Equation

For the solid fractions of 2.5%, 7.4%, 14.5%, and 22.7% with 40 μm height, the calculated Cassie's water contact angles using **Eq. 4** and the measured advancing contact angle on the hydrophobic reference Si surface (i.e., 110°), are 169, 162, 155, and 148°, respectively. The measured advancing contact angles are 167, 169, 166, and 169°, and the receding contact angles are 159, 148, 137, and 131°, respectively, showing similar trends between the calculated and measured values. The receding contact angle in **Figure 4f** is decreasing as solid fraction is increasing as suggested by Cassie-Baxter equation. This is due to higher adhesion

forces, for the same droplet area, as the solid-liquid interface is increasing in the greater solid fractions.

2.5. Marmur Minimum Roughness

As per Marmur's findings, when r decreases below the minimum roughness threshold r_{min}, the Cassie state durability tends to get shorter. This aligns seamlessly with our experimental outcomes regarding pillar size, solid fraction, and pillar height effects on plastron lifetime. Notably, the [10X20X40 µm-**SHB-8**] surface, features the 10 µm pillars with the greatest roughness ratio r of 4.63 (calculated by SEM measured structure dimensions), significantly surpassing its corresponding calculated r_{min} (2.49), suggesting a great stability when submerged in a fluid. Our results affirm this observation with the biofluid used. Conversely, the [10X60X15 µm-**SHB-1**] surface with an r value of 1.15, significantly below the required r_{min} (2.88) for a stable underwater Cassie state, experiences a rapid transition from Cassie to Wenzel state upon immersion in the biofluid. Meanwhile, the [2X4X30 µm] surface exhibits an exceptionally high r value of 12.78, indicating substantial underwater stability. However, its mechanical robustness is very poor due to pillar aspect ratio of 15, making it hard to be fabricated without defects and impossible to last long under harsh conditions. Consequently, in the design of superhydrophobic surfaces for under_fluid applications, careful consideration of each parameter impact on plastron lifetime is crucial, and it is important to recognize the limiting factors such as r_{min} and mechanical robustness.

REFERENCES

(1) Mohammadi, R.; Wassink, J.; Amirfazli, A. Effect of Surfactants on Wetting of Super-Hydrophobic Surfaces. *Langmuir* 2004, *20* (22), 9657–9662. https://doi.org/10.1021/la049268k.

(2) Accardo, A.; Gentile, F.; Mecarini, F.; De Angelis, F.; Burghammer, M.; Di Fabrizio, E.; Riekel, C. In Situ X-Ray Scattering Studies of Protein Solution Droplets Drying on Micro-and Nanopatterned Superhydrophobic PMMA Surfaces. *Langmuir* 2010, *26* (18), 15057–15064. https://doi.org/10.1021/la102958w.

(3) Choi, C. H.; Kim, C. J. Droplet Evaporation of Pure Water and Protein Solution on Nanostructured Superhydrophobic Surfaces of Varying Heights. *Langmuir* 2009, *25* (13), 7561–7567. https://doi.org/10.1021/la803614h.

3. Supplementary Tables

Table S1. The Si micropillars geometry parameters that were used in this study.

**Pillar size [μm]**	**Solid fractions [%]**	**Pillar height [μm]**	**Total number of surfaces**
2, 5, 10, 20, 30, 40, and 50	2.5, 7.4, 14.5, 19.6, and 22.7	3, 10, 15, 30, and 40	More than 50

Table S2. The prepared biofluids in this study. Abbreviations and compositions.

**Biofluid**	**Content**
**RPMI 1640**	RPMI 1640 cell medium with no supplements
**BSA solutions**	2, 5, 8, 20, 50, or 100 mg mL^{-1} in DI water
**1.25Glu**	1.25 mg mL^{-1} glucose in RPMI 1640 cell media
**10%FBS**	10% FBS in RPMI 1640 cell medium
**50%FBS**	50% FBS in RPMI 1640 cell medium
**100%FBS**	FBS only
**8BSA+0.125Glu**	8 mg mL^{-1} of BSA and 0.125 mg mL^{-1} glucose in RPMI 1640 cell media
**50BSA+10%FBS**	50 mg mL^{-1} oof BSA and 10% FBS in RPMI 1640 cell media
**80BSA+1.25Glu**	80 mg mL^{-1} of BSA and 1.25 mg mL^{-1} glucose in RPMI 1640 cell media
**80BSA+2Glu**	80 mg mL^{-1} of BSA and 2 mg mL^{-1} glucose in RPMI 1640 cell media
**100BSA+2Glu+100%FBS**	100 mg mL^{-1} of BSA and 2 mg mL^{-1} glucose in FBS

4. Supplementary Figures

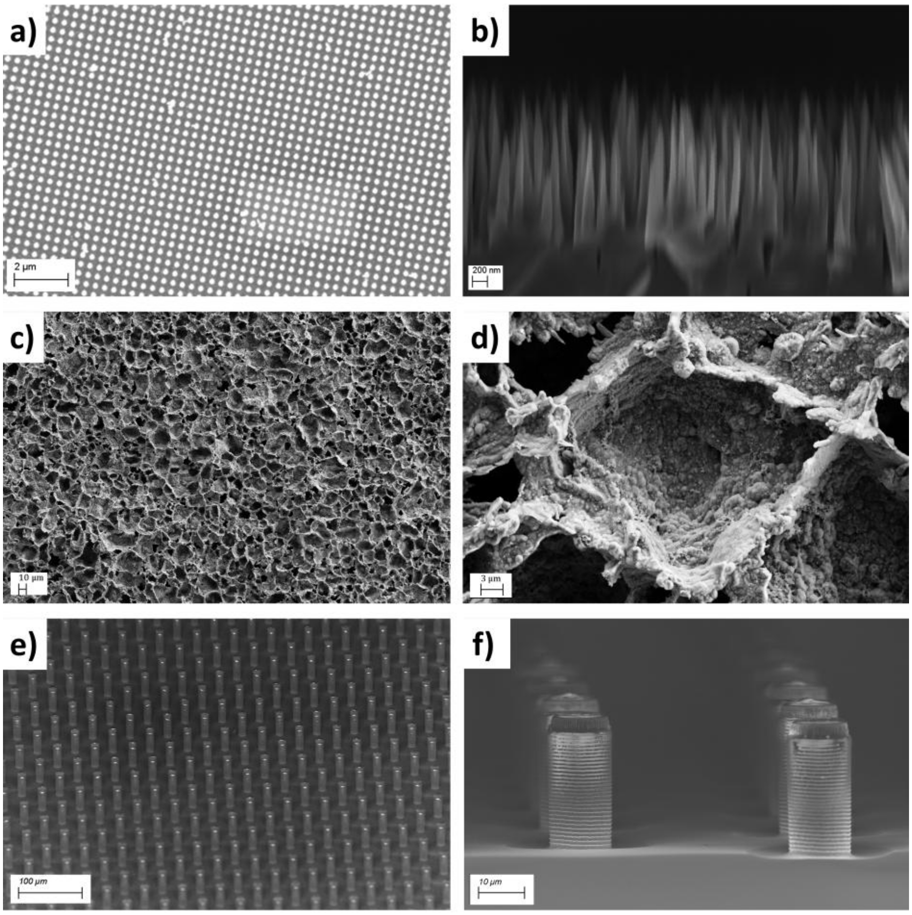

Figure S1. SEM images of **a)** Si nanopillars. **b)** black Si. **c)** and **d)** Cu-PDMS. **e)** and **f)** Si micropillars.

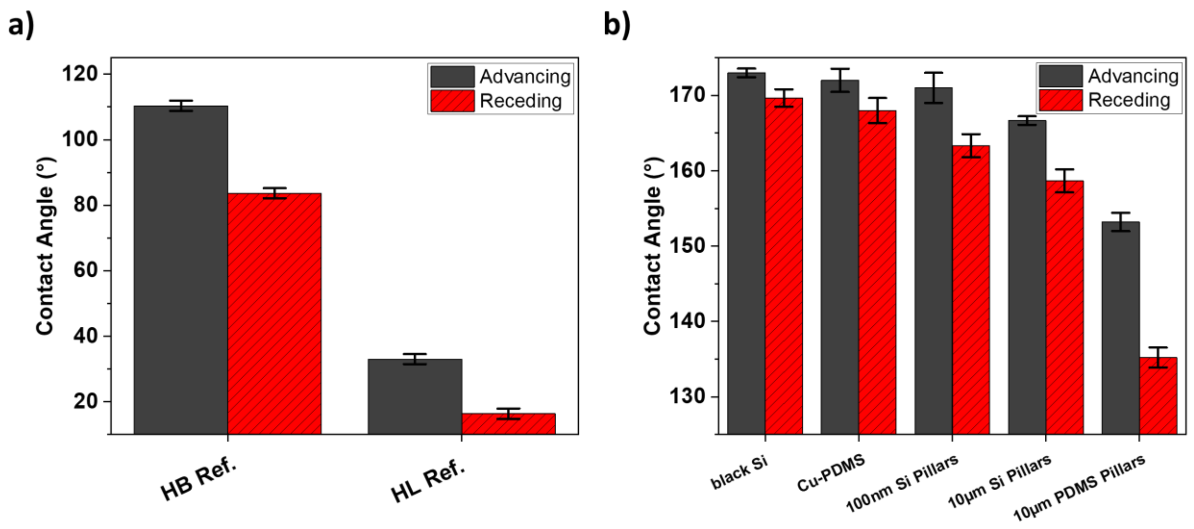

Figure S2. The water advancing and receding contact angles of the studied surfaces. **a)** The hydrophobic (HB Ref) and hydrophilic (HL Ref) smooth Si reference surfaces. **b)** Black Si, Cu-PDMS, 100 nm Si pillars, Si 10 μm pillars with 2.5% solid fraction and 40 μm pillar depth, and PDMS 10 μm pillars with 22.7% solid fraction and 40 μm pillar depth.

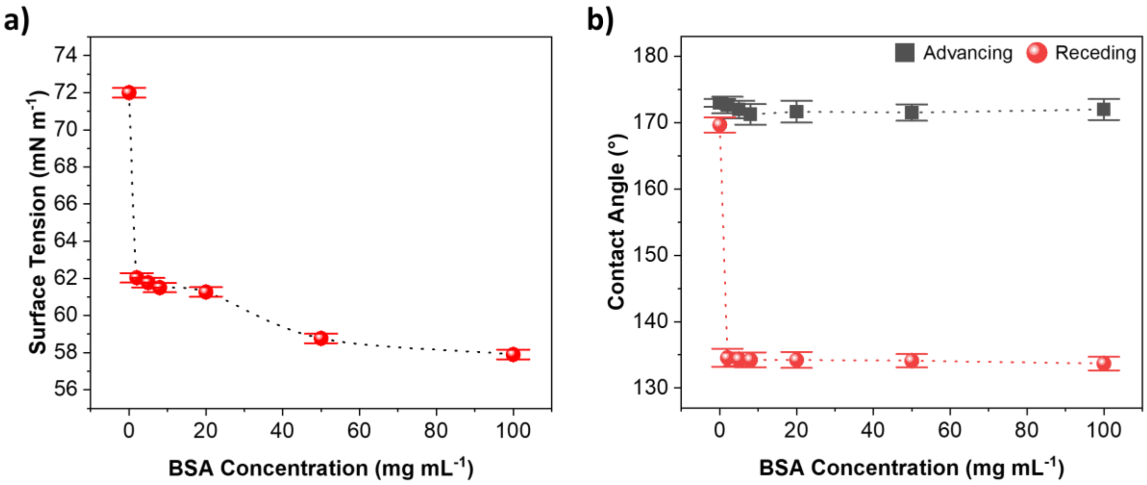

Figure S3. a) The surface tensions of BSA solutions with different concentrations (0, 2, 5, 8, 20, 50, and 100 mg mL^{-1}). b) The dynamic contact angles of water and the six BSA concentrations solutions on Black Si SHB surface.

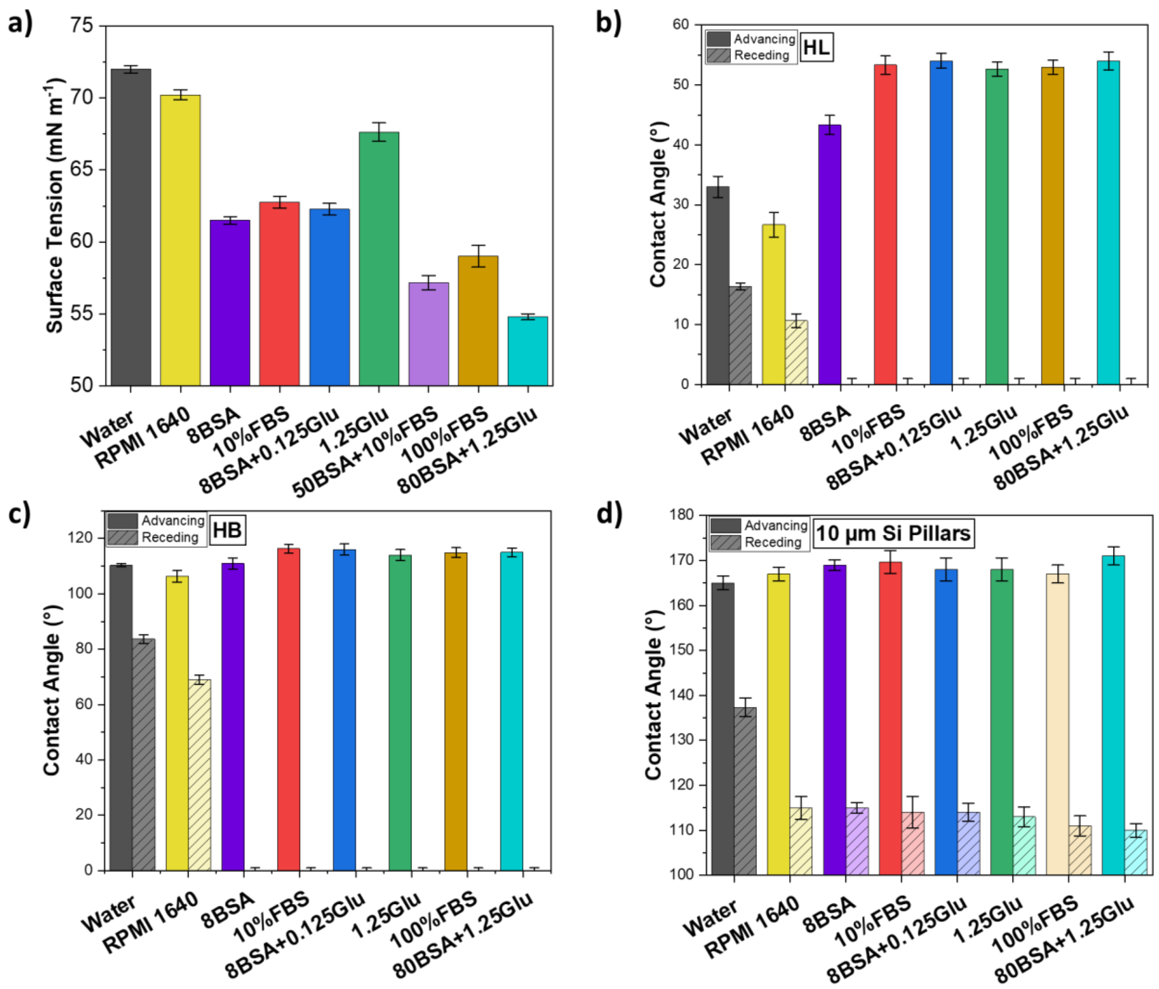

Figure S4. Surface tensions and dynamic contact angles of biofluids on a fluoropolymer hydrophobic coating. **a)** Surface tension measurements of different biofluids. The dynamic contact angles of the biofluids on **b)** hydrophilic reference surface (HL), **c)** hydrophobic reference surface (HB), and **d)** SHB 10 μm Si pillared superhydrophobic surface with 14.5% solid fraction and 15 μm height.

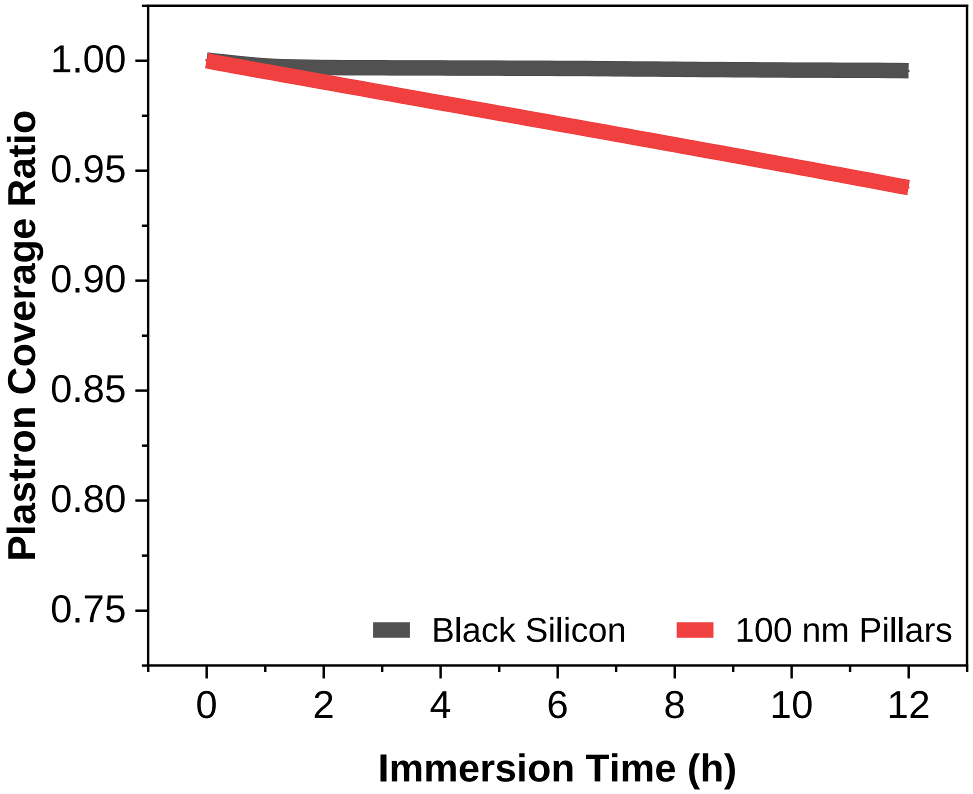

Figure S5. Plastron lifetime of black Si and 100 nm Si pillared SHB surfaces in RPMI 1640 medium containing 2 mg mL^{-1} of BSA.

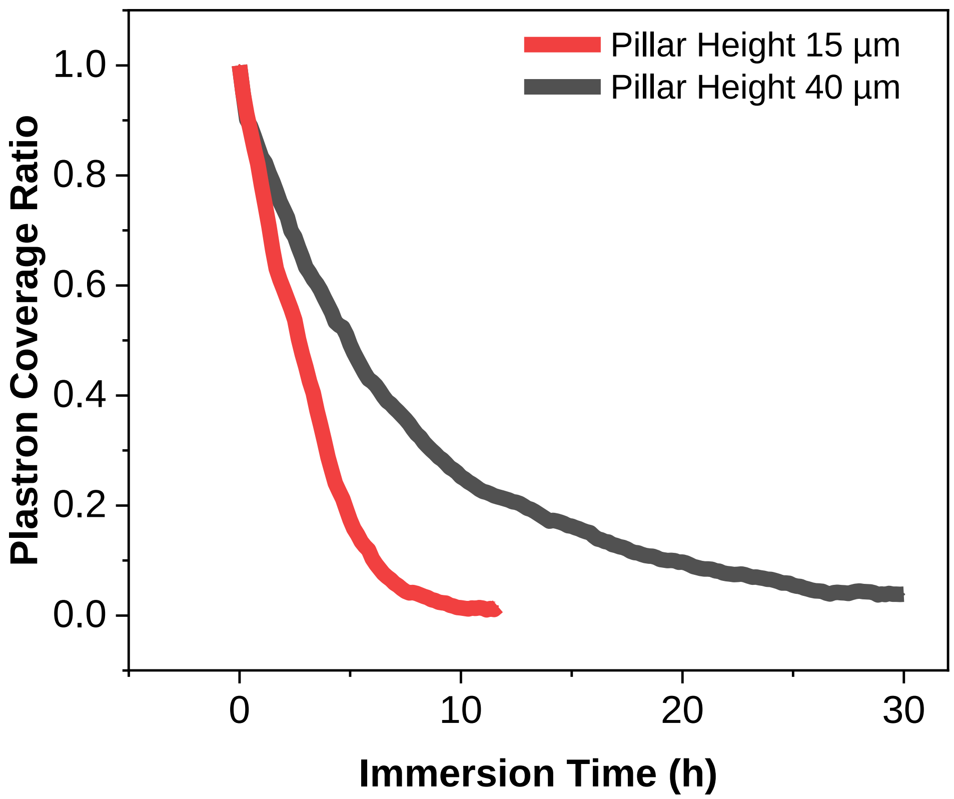

Figure S6. The effect of pillar height on plastron lifetime. The plastron coverage ratio over time of Si pillars, with dimensions of 10 μm pillar size and 22.7% solid fraction and with two pillar height (15 and 40 μm), immersed in FBS serum supplemented with 100 mg mL^{-1} of BSA and 2 mg mL^{-1} of glucose.

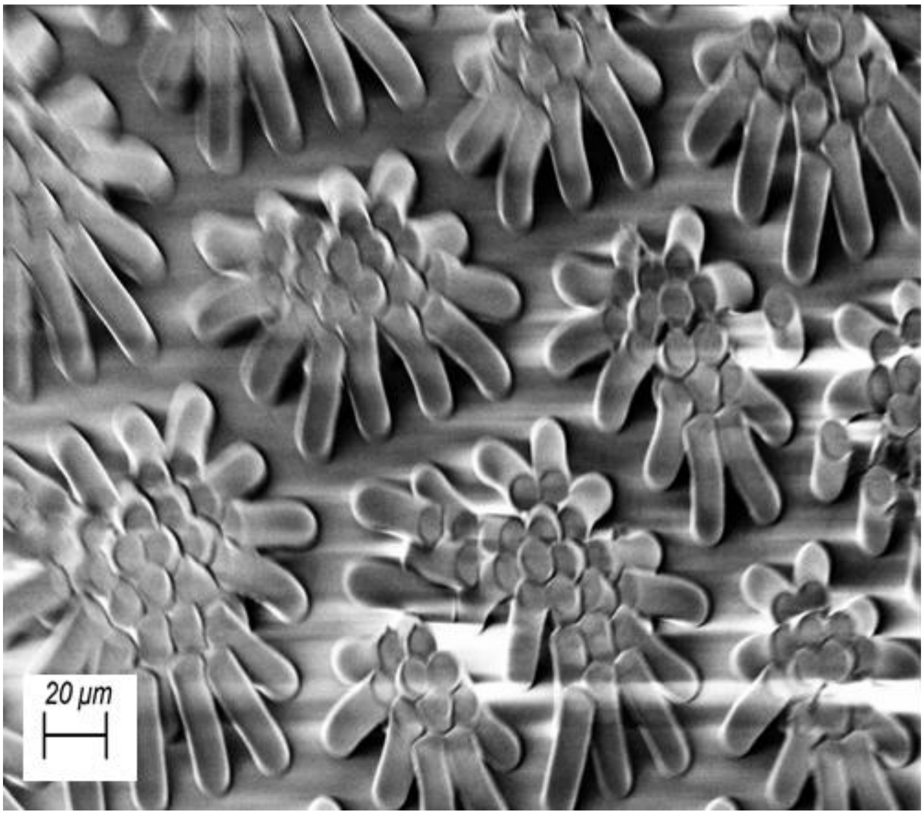

Figure S7. SEM image of PDMS pillar sticking to each other after their immersion in a biofluid.

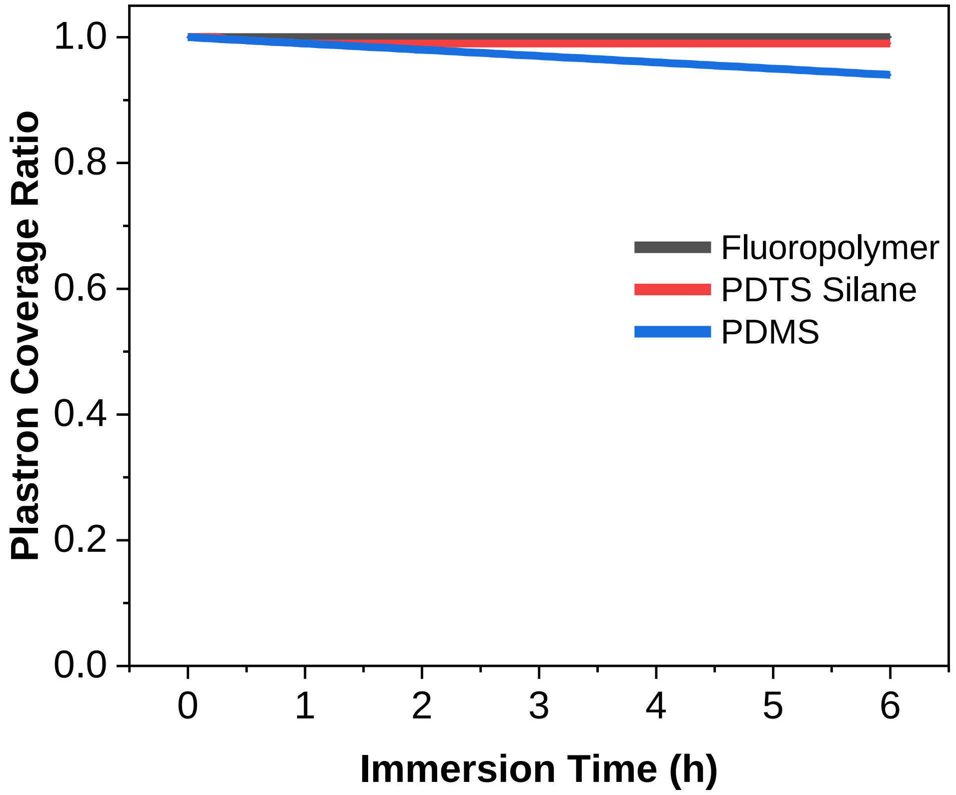

Figure S8. Surface chemistry effect on plastron lifetime underwater.

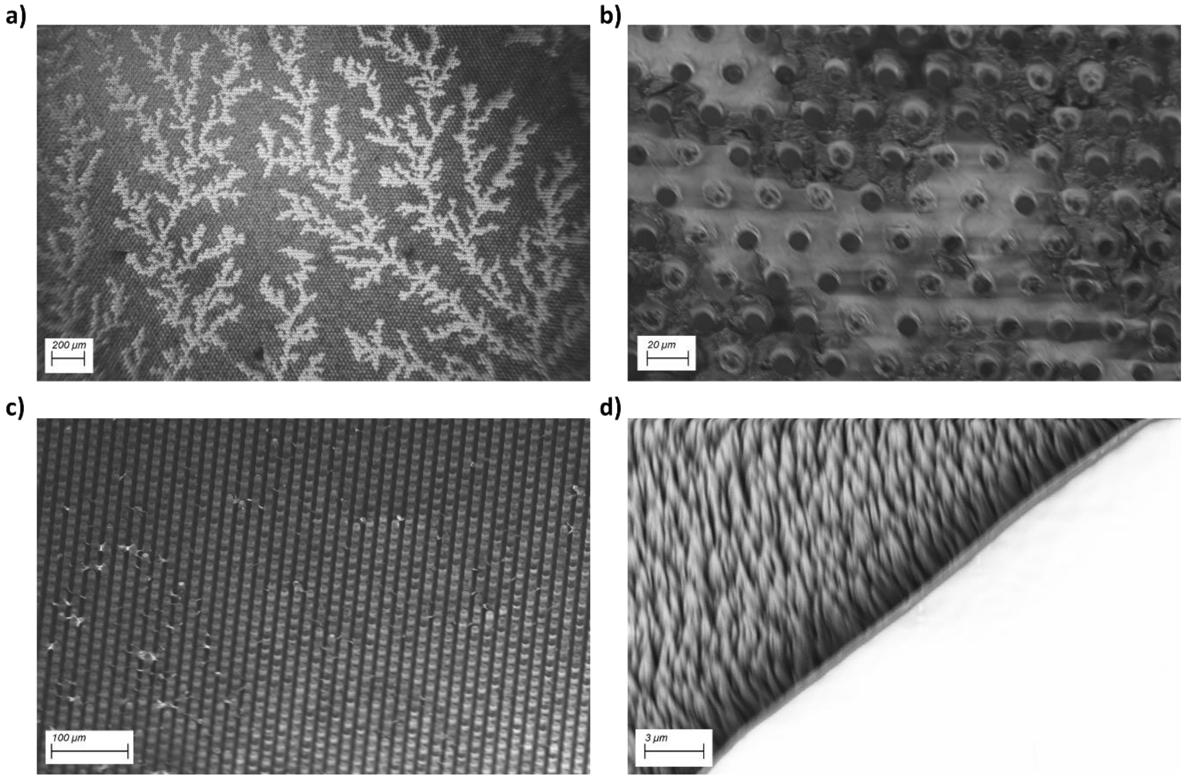

Figure S9. SEM images of **a)** Si micropillared surface immersed in a protein solution for a week. The brighter regions had a Wenzel state for a few days, while the darker regions had a Wenzel state only for hours. **b)** a closer look on the image in (a). **c)** Si micropillared surface immersed in a protein solution for several days and retained its Cassie state. The protein agglomerates are shown on top of the pillars. **d)** black Si surface have immersed in a protein solution for a few weeks. The bright white film is a thick deposited biofilm over the black Si, while the dry black Si out of the solution is shown on the left side.

5. Supporting Videos Description

Supp. Video 1.

The video demonstrates the bubble mechanism by showing the plastron gradual dissipation on a Si 10 µm pillared superhydrophobic surface (Pillar Height 15 µm, Solid Fraction 22.7%) immersed in a biofluid. Note the big air bubbles that grow and detach from the surface. The time scale of this video is 120 h.

Supp Video 2.

The video shows the air film rupturing of a Si 10 µm pillared superhydrophobic surface (Pillar Height 40 µm, Solid Fraction 22.7%) after being immersed in a complex biofluid for more than a week. The surface was left mostly dry indicating a Cassie state.
